# Structure-guided steric hindrance engineering of *Bacillus badius* phenylalanine dehydrogenase for efficient l-homophenylalanine synthesis

**DOI:** 10.1186/s13068-021-02055-0

**Published:** 2021-10-24

**Authors:** Tao Wu, Xiaoqing Mu, Yuyan Xue, Yan Xu, Yao Nie

**Affiliations:** 1 Laboratory of Brewing Microbiology and Applied Enzymology, School of Biotechnology, Jiangnan University, Wuxi, 214122 China; 2 Key Laboratory of Industrial Biotechnology, Ministry of Education, School of Biotechnology, Jiangnan University, Wuxi, 214122 China; 3 Suqian Jiangnan University Institute of Industrial Technology, Suqian, 223800 China

**Keywords:** l-Homophenylalanine, Phenylalanine dehydrogenase, Steric hindrance, Enzyme engineering, Catalytic efficiency, Reductive amination

## Abstract

**Background:**

Direct reductive amination of prochiral 2-oxo-4-phenylbutyric acid (2-OPBA) catalyzed by phenylalanine dehydrogenase (PheDH) is highly attractive in the synthesis of the pharmaceutical chiral building block l-homophenylalanine (l-HPA) given that its sole expense is ammonia and that water is the only byproduct. Current issues in this field include a poor catalytic efficiency and a low substrate loading.

**Results:**

In this study, we report a structure-guided steric hindrance engineering of PheDH from *Bacillus badius* to create an enhanced biocatalyst for efficient l-HPA synthesis. Mutagenesis libraries based on molecular docking, double-proximity filtering, and a degenerate codon significantly increased catalytic efficiency. Seven superior mutants were acquired, and the optimal triple-site mutant, V309G/L306V/V144G, showed a 12.7-fold higher *k*_cat_ value, and accordingly a 12.9-fold higher *k*_cat_/*K*_m_ value, than that of the wild type. A paired reaction system comprising V309G/L306V/V144G and glucose dehydrogenase converted 1.08 M 2-OPBA to l-HPA in 210 min, and the specific space–time conversion was 30.9 mmol g^−1^ L^−1^ h^−1^. The substrate loading and specific space–time conversion are the highest values to date. Docking simulation revealed increases in substrate-binding volume and additional degrees of freedom of the substrate 2-OPBA in the pocket. Tunnel analysis suggested the formation of new enzyme tunnels and the expansion of existing ones.

**Conclusions:**

Overall, the results show that the mutant V309G/L306V/V144G has the potential for the industrial synthesis of l-HPA. The modified steric hindrance engineering approach can be a valuable addition to the current enzyme engineering toolbox.

**Supplementary Information:**

The online version contains supplementary material available at 10.1186/s13068-021-02055-0.

## Background

l-Homophenylalanine (l-HPA) is an unnatural aromatic amino acid and contains a single-carbon extended side chain compared with l-phenylalanine [1]. l-HPA can serve as a versatile chiral building block for the synthesis of several blockbuster pharmaceuticals, such as angiotensin-converting enzyme inhibitors [2, 3], proteasome inhibitors [4, 5], acetylcholinesterase inhibitors [6], and β-lactam antibiotics [6]. Chemical methods of l-HPA synthesis are constrained by lengthy steps, high cost, environmental pollution, and poor enantioselectivity, rendering industrial processes ultimately unsustainable [7, 8]. By contrast, biocatalysts are environmentally friendly, mild reaction conditions, and have excellent selectivity [9, 10]. Thus, various enzymatic routes including kinetic resolution catalyzed by lipase [11], in situ racemization catalyzed by hydantoinase [12, 13], and asymmetric transamination catalyzed by transaminase [14, 15] were recently developed and applied to l-HPA synthesis. Nevertheless, the foregoing reactions also have limitations, such as a theoretical maximum 50% lipase yield, poor applicability because of the strict d-enantioselectivity of hydantoinase, and undesirable equilibrium shifts in the reversible transaminase transamination [16].

**Fig. 1:**
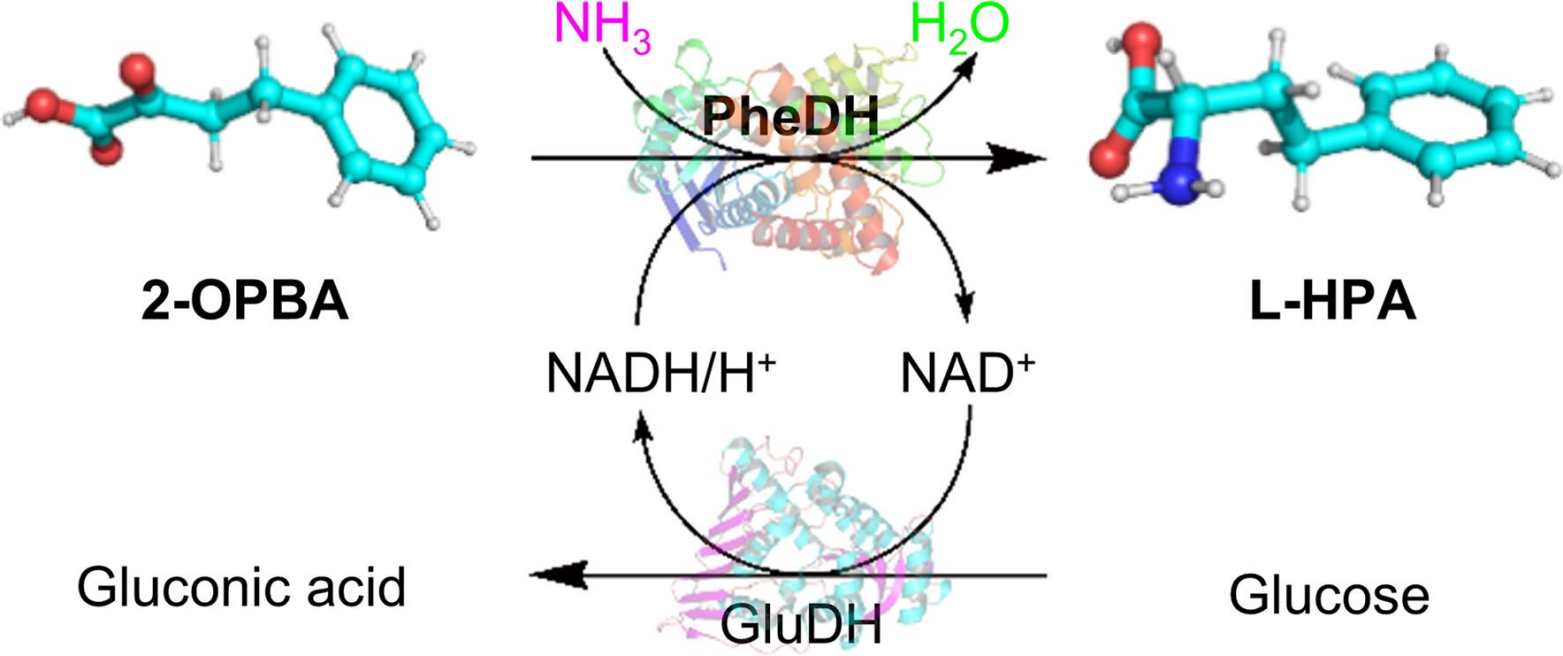
Biosynthesis of l-HPA from 2-OPBA catalyzed by PheDH paired with a GluDH cofactor recycling system

Direct asymmetric reductive amination of prochiral 2-oxo-4-phenylbutyric acid (2-OPBA) catalyzed by phenylalanine dehydrogenase (PheDH) is a promising approach in l-HPA synthesis. It consumes only free ammonia and generates only water as a byproduct (Fig. 1). Asano et al. [17] reported the use of PheDH from *Bacillus sphaericus* (*Bs*PheDH) to prepare l-HPA. Bradshaw et al. [18] investigated the application of recombinant *E. coli* cells harboring PheDH from *Rhodococcus* sp. M4 (*Rs*PheDH) to produce l-HPA. They achieved 63% conversion using 60 mM substrate. Ahmad et al. [19] enhanced the applicability of *Rs*PheDH in l-HPA production via cell immobilization. They achieved > 80% conversion using 67 mM substrate. Notably, Zhang et al. [20] stated that the combination of substrate fed-batch with continuous product removal improved substrate loading in l-HPA production catalyzed by PheDH from *Thermoactinomyces intermedius* (*Ti*PheDH). A final substrate concentration of 510 mM was achieved after eight cycles of a fed-batch operation. An important constraint in PheDH-catalyzed l-HPA production is the poor catalytic efficiency toward non-native, bulky 2-OPBA compared with native phenylpyruvic acid (PPA). Consequently, the substrate loading is low and fails to meet the requirements of industrially and commercially viable biocatalysts and biocatalysis processes [21].

Directed evolution techniques are powerful tools for generating prolific sources of biocatalysts [22, 23]. Steric hindrance of the substrate-binding pocket plays a significant role in modulating the catalytic properties of an enzyme, including enantioselectivities [24, 25], substrate specificities [26–28], and catalytic activities [25, 28–30]. Recent enzyme engineering studies focused on increasing or conferring enzyme activity towards non-native bulky substrates by attenuating the steric hindrance of the substrate-binding pocket. The mutant constructed by Merck and Codexis [31] using transaminase ATA-117 as a mutagenic template is a remarkable example of such engineering, as the mutant successfully showed detectable reductive amination activity toward the bulky prositagliptin ketone that lacked any activity before, which was successfully used to catalyze the synthesis of the antidiabetic compound sitagliptin. Chen et al. [32] used rational design based on steric hindrance attenuation to induce asymmetric reductive amination activity of engineered amine dehydrogenases towards several previously unreactive long-chain α-aliphatic ketones. The hydroxynitrile lyase mutants engineered by Zheng et al. [33] showed remarkably increased asymmetric hydrocyanation activity toward the rigid pharmaco aldehydes, and successfully applied them to achieve the synthesis of the β_2_-adrenoreceptor agonist salmeterol. These successful advances demonstrated the feasibility of steric hindrance engineering of substrate-binding pockets to modulate the catalytic activity of enzymes towards non-native, bulky substrates.

In the present study, we report the structure-guided steric hindrance engineering of PheDH from *Bacillus badius* (*Bb*PheDH) to improve its catalytic efficiency in l-HPA synthesis. The steric hindrance of the substrate-binding pocket of *Bb*PheDH was subjected to crucial residue identification and evaluation of the mutagenesis libraries constructed based on degenerate codons. Seven superior mutants were generated after four rounds of steric hindrance engineering. The optimal triple-site mutants significantly enhanced catalytic efficiency, substrate loading, and specific space–time conversion. Structure analysis elucidated the factors contributing to the enhanced catalytic efficiency observed in the enzyme-kinetics and transformation experiments.

## Results and discussion

### Preliminary activity studies and molecular modeling

Initially, two PheDHs from *B. badius* (*Bb*PheDH) and *G. kaustophilus* (*Gk*PheDH) were selected as candidate enzymes in the development of an enhanced biocatalyst for l-HPA synthesis. As 2-OPBA had a larger side chain than the native substrate PPA, its catalysis by *Bb*PheDH and *Gk*PheDH was characterized by lower turnover frequency (*k*_cat_) and less favorable catalytic efficiency (*k*_cat_/*K*_m_) (Table 1), which motivated us to consider enhancing catalytic efficiency toward 2-OPBA by the steric hindrance engineering. Biotransformation experiments were subsequently performed using *Bb*PheDH and *Gk*PheDH as biocatalysis, giving 67.5% and 53.6% conversions at 0.2 M substrate concentration within 8 h (Table 1 and Additional file 2: Figure S1). Due to its relatively higher conversion, *Bb*PheDH was selected as the starting mutagenesis template for steric hindrance engineering.

**Table 1:** Kinetic parameters of *Bb*PheDH and *Gk*PheDH toward PPA and 2-OPBA and conversions of *Bb*PheDH and *Gk*PheDH in the reductive amination of 2-OPBA

Enzyme	Substrate	*k*_cat_ (s^−1^)	*K*_m_ (mM)	*k*_cat_/*K*_m_ (mM^−1^ s^−1^)	Conversion (%)
*Bb*PheDH	PPA	115 ± 6	0.3 ± 0.04	370 ± 45	–
	2-OPBA	11 ± 1	0.6 ± 0.06	17 ± 1	67.5
*Gk*PheDH	PPA	92 ± 6	0.3 ± 0.04	340 ± 30	–
	2-OPBA	6 ± 1	0.5 ± 0.04	11 ± 2	53.6

**Fig. 2:**
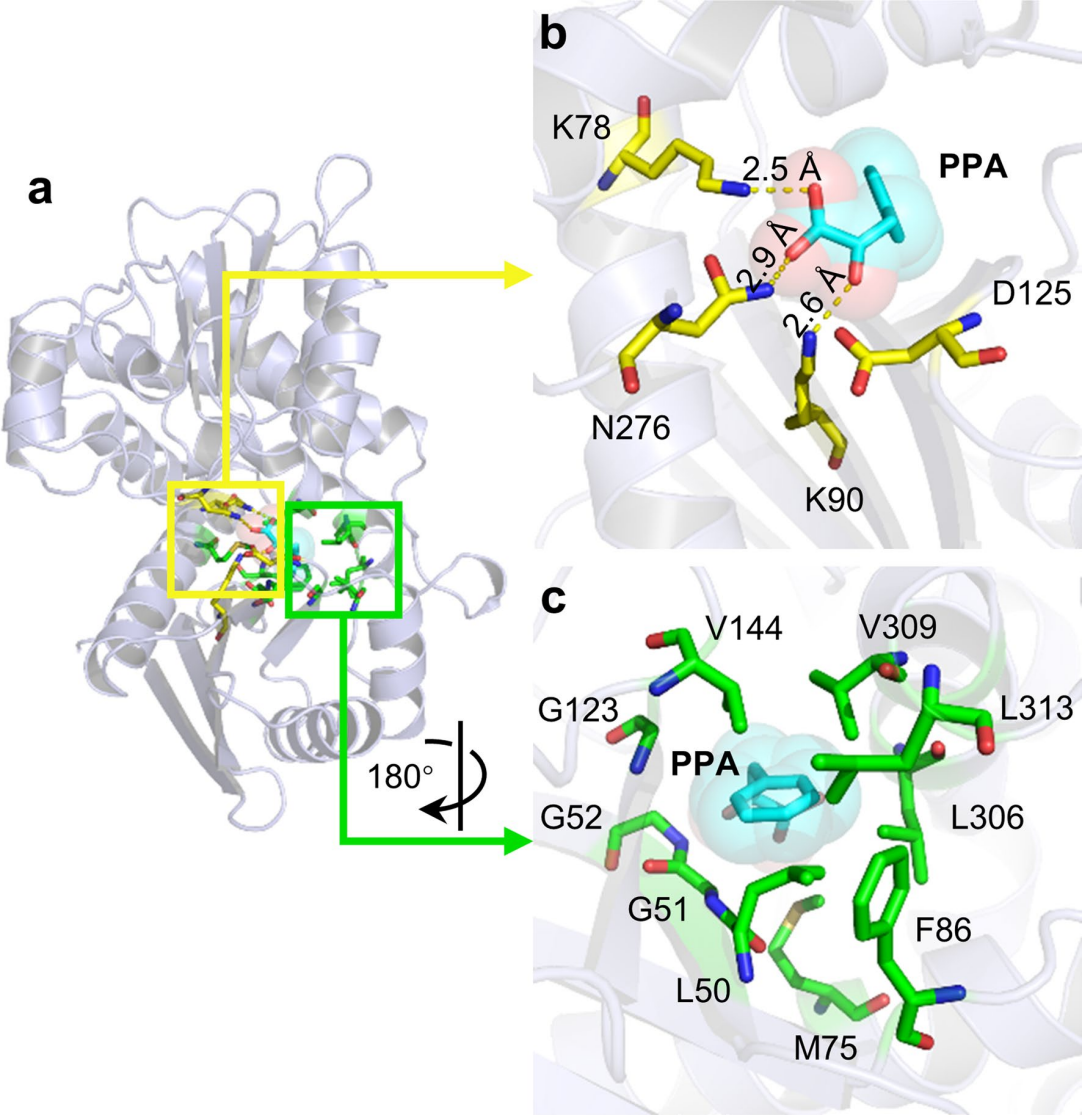
Structural homology model of *Bb*PheDH. Complex model of *Bb*PheDH and the native substrate PPA (**a**) and corresponding hydrophilic (**b**) and hydrophobic (**c**) areas in substrate-binding pocket of *Bb*PheDH. Structural homology model of enzyme is shown in light blue cartoon. Substrate PPA is depicted as cyan sticks and balls. Catalytic residues K78, K90, D125, and N276 are shown as yellow sticks. Hydrophobic residues L50, G51, G52, M75, F86, G123, V144, L306, V309, and L313 are shown as green sticks

To assess the feasibility of developing an enhanced biocatalyst for efficient l-HPA synthesis, we first generated a structural homology model of *Bb*PheDH (Fig. 2a) based on the reported crystal structure of *Rhodococcus* sp. M4 PheDH [34] to develop hypotheses for mutagenesis library designs. The model revealed that the residues surrounding the catalytic active center of *Bb*PheDH could be classified into two categories on the basis of their function in the substrate-binding process. Group one encompasses the catalytic residues that are appointed in the binding of the α-carboxylic and α-amino groups of the substrate, namely, K78, K90, D125, and N276, respectively, which were highly conserved among the PheDH family (Additional file 2: Figure S2). These residues create a hydrophilic environment that combines the hydrophilic α-amino acid moiety of the substrate (Fig. 2b). On the opposite side, a panel of hydrophobic residues creates a hindered hydrophobic environment forcing the substrate side chain into its ideal binding pose (Fig. 2c). Furthermore, limited structure-guided engineering was previously conducted on PheDHs and showed that most of the beneficial mutations introduced into the substrate-binding pocket were hydrophobic amino acid residues [35–37]. Based on the foregoing information, we selected amino acids with strong hydrophobicity and weak steric hindrance as target substitutes in the subsequent mutagenesis experiments.

### Mutagenesis library construction and evaluation based on attenuated steric hindrance

**Fig. 3:**
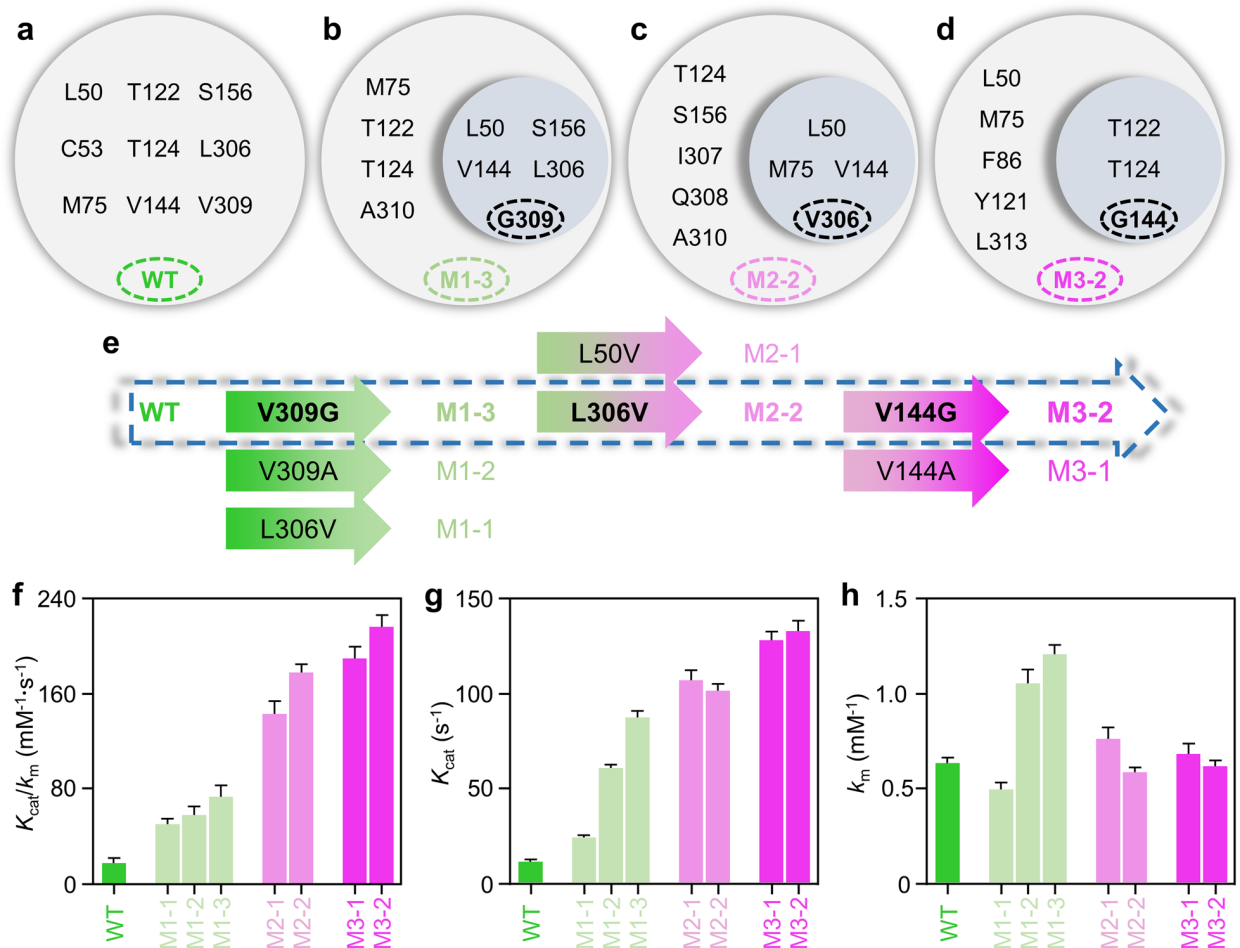
Screening and characterization of superior mutants. Residues selected for steric hindrance mutagenesis in wild-type *Bb*PheDH (**a**), and mutant M1–3 (**b**), M2–2 (**c**), and M3–2 (**d**). Screening of superior mutants (**e**) and kinetic parameters of *Bb*PheDH and superior mutants (**f**–**h**). Residues shown in gray bubbles, including in blue bubbles (**a**–**d**), were all close (within 6 Å) to the side-chain phenyl ring of 2-OPBA, while the residues in blue bubbles (**b**–**d**) are not only close to the side-chain phenyl ring of 2-OPBA, but also close to the focal residues (G309, V306, and G144, respectively). Activity was measured in NH_4_Cl/NH_4_OH buffer (2 M, pH 9.5) containing 1–20 mM 2-OPBA and 0.5 mM NADH at 30 °C and carried out at a 200-μL scale in 96-well microtiter plates by monitoring the initial decrease velocity of the absorbance at 340 nm (indicating NADH consumption). All determinations were performed in triplicate, and error bounds represent ± sd

To identify mutagenesis candidates for initial library construction, 2-OPBA was docked into the catalytic active center of *Bb*PheDH to investigate the unfavorable hindrance interactions. Based on the acquired binding pose, all amino acid residues except the smallest glycine and the conserved catalytic residues within 6 Å of the side-chain phenyl ring of 2-OPBA were selected for site-directed mutagenesis in the first round of steric hindrance engineering (Fig. 3a). Twenty-three single-site mutants were constructed at nine amino acid sites based on the designed degenerate codon (Additional file 1: Table S1) by whole-plasmid PCR [38]. All target mutants were isolated from the mixed libraries and confirmed by DNA sequencing. Three superior mutants L50V (M1–1), V309A (M1–2), and V309G (M1–3) were identified by initial activity screening of cell-free extracts (Additional file 2: Figure S3). Subsequent enzyme-kinetics experiments with the purified proteins demonstrated that the *K*_m_ value of these mutants toward 2-OPBA did not change significantly (Fig. 3h), whereas the *k*_cat_ values were 25, 61, and 89 s^−1^, which were 2.3-, 5.7-, and 8.3-fold higher than that of the wild type (11 s^−1^), respectively (Fig. 3g).

As mutant M1–3 had the highest catalytic efficiency (74 s^−1^ mM^−1^) towards 2-OPBA, it was selected as the mutagenesis template in the second round of steric hindrance engineering to investigate the potential synergistic effects. The binding pose of M1–3 with 2-OPBA indicated eight amino acid residues within 6 Å of the side-chain phenyl ring of 2-OPBA. For the rapid detection of any synergy of a proximal steric hindrance with residue G309, the residues were simplified by double-proximity filtering. We identified four mutagenesis candidates within 6 Å of both the phenyl ring of 2-OPBA and G309 (Fig. 3b). Thus, a mutagenesis library consisting of ten double-site mutants was constructed, and the superior mutants V309G/L50V (M2–1) and V309G/L306V (M2–2) were identified by the initial activity screening of cell-free extracts (Additional file 2: Figure S4). The *k*_cat_/*K*_m_ values of the purified mutant proteins, M2–1 and M2–2, toward 2-OPBA were determined to be 143 and 179 s^−1^ mM^−1^, respectively, which were 8.4- and 10.5-fold higher than that of the wild type, respectively (Fig. 3f). Notably, this change was also caused by the enhanced *k*_cat_ value of the mutants (Fig. 3g).

With this result, the mutant M2–2 was selected as the template in the third round of steric hindrance engineering. Here, we investigated proximal steric hindrance synergy with V306. Three mutagenesis candidates were identified by double-proximity filtering using V306 as the key residue (Fig. 3c). The initial activity screening of the cell-free extracts of the ten triple-site mutants revealed two superior mutants V309G/L306V/V144A (M3–1) and V309G/L306V/V144G (M3–2) (Additional file 2: Figure S5). The *k*_cat_ values of the purified mutant proteins, M3–1 and M3–2, toward 2-OPBA reached 131 and 136 s^−1^, respectively, which were 12.2- and 12.7-fold higher than that of the wild type, respectively (Fig. 3g); this change was accompanied by a 11.3- and 12.9-fold increase in the *k*_cat_/*K*_m_ values, respectively (Fig. 3f).

Inspired by the above results, we explored the possibility of increasing catalytic activity towards 2-OPBA using M3–2 as the template in the fourth round of steric hindrance engineering. Two mutagenesis candidates were identified (Fig. 3d) and four corresponding quadruple-site mutants were constructed. However, none of them exhibited higher activity towards 2-OPBA than M3–2 (Additional file 2: Figure S6).

Together, seven superior mutants were successfully screened from the 47 elements in the site-directed mutagenesis library (Fig. 3e). The optimal triple-site mutant, M3–2 (V309G/L306V/V144G), showed a 12.7-fold increase in the *k*_cat_ value (Fig. 3g) compared with that of the wild-type *Bb*PheDH, whereas no obvious change was observed in the *K*_m_ value (Fig. 3h), which resulted in a 12.9-fold increase in the *k*_cat_/*K*_m_ value (Fig. 3f). This result was similar to that of other studies [25, 32]. Subsequently, the kinetic parameters of *Bb*PheDH and obtained mutants with superior activity toward NADH were assessed. The results showed that the catalytic efficiency of the mutants toward NADH was improved to varying degrees, which is beneficial for the practical application of these mutants to l-HPA synthesis. Moreover, the thermostabilities of *Bb*PheDH and its superior mutants were assessed by measuring the *T*_50_^30^ value (the temperature at which 50% of enzyme activity is lost following a heat treatment for 30 min). It is not unexpected that some kind of stability-activity tradeoff occurred in all mutants, although this tradeoff is of no practical importance, since the *T*_50_^30^ value of all mutants still exceeded 50 ℃ (Additional file 1: Table S2).

### ***Biocatalytic optimization analysis on the best mutant M3***–***2***

**Fig. 4:**
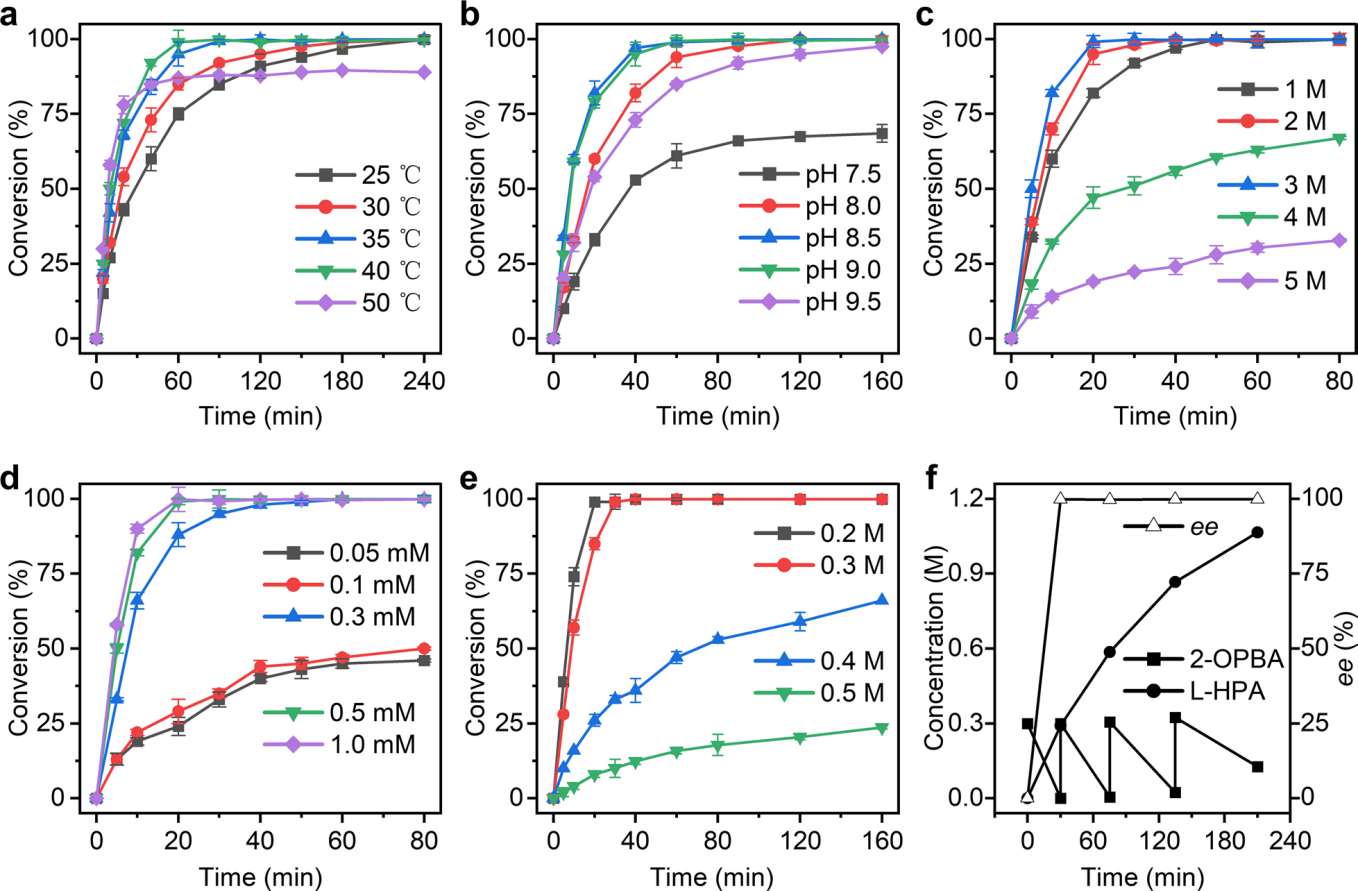
Optimization studies and reductive amination employing mutant M3-2. Influences of temperature (**a**), pH (**b**), NH_4_^+^ concentration (**c**), and NAD^+^ concentration (**d**) on the reductive amination of 2-OPBA using M3–2. Reductive amination of 2-OPBA in presence of 0.2–0.5 M substrate (**e**) and application of a combination of substrate fed-batch with product removal (**f**). Conversion of 2-OPBA was determined by monitoring the concentration of 2-OPBA in the reaction mixture with HPLC analysis. Absolute configuration and ee value of l-HPA were established and calculated by comparing the obtained values with those from authentic reference material after derivatization. The biotransformation experiment was performed in triplicate, and error bounds represent ± sd

The dependence of temperature and pH value were investigated for the reductive amination of 2-OPBA catalyzed by M3–2 paired with glucose dehydrogenase (GluDH) at 0.2 M substrate concentration. Figure 4a shows that 2-OPBA conversion was > 99% after 180 min when the reactions proceeded at 30–40 °C. Catalytic efficiency decreased slightly at 25 °C, with the reaction requiring 240 min to attain 99% conversion. At 50 °C, however, quantitative conversion was < 90% after 240 min. We then evaluated the effects of NH_4_OH/HCOONH_4_ buffer (1 M) at various pH values and 30 °C. Figure 4b shows that the fastest conversions occurred at pH 8.5 and the quantitative conversion was > 99% within 90 min. We subsequently performed the reductive amination of 2-OPBA in various concentrations of NH_4_OH/HCOONH_4_ buffer (pH 8.5) and NAD^+^ to determine the influences of NH_4_^+^ and NAD^+^ concentration. The 2-OPBA conversion was > 99% at 1–3 M NH_4_^+^. By contrast, 4 M and 5 M NH_4_^+^ lowered the conversion rate (Fig. 4c). An activity assay indicated that low GluDH activity might explain the observed decrease in conversion rate in the presence of high NH_4_^+^ concentrations (Additional file 2: Figure S7). Moreover, 2-OPBA could be fully converted when the NAD^+^ concentration was 0.3 mM, 0.5 mM, or 1 mM; however, the conversion dropped to < 50% when the NAD^+^ concentration was < 0.3 mM (Fig. 4d).

### Preparative scale synthesis of l-HPA employing mutant M3–2

Using the optimized reaction system (30 °C; pH 8.5; 3 M NH_4_^+^; 0.3 mM NAD^+^), the preparative scale synthesis of l-HPA by reductive amination of 2-OPBA was carried out in 100 mL reaction volume. The pH value was maintained at 8.5 by titrating concentrated NH_3_·H_2_O during the reaction course. Figure 4e shows that 0.2 M and 0.3 M 2-OPBA were converted by > 99% conversion within 20 min and 30 min, respectively. However, substrate inhibition was observed at high concentrations. The quantitative conversion rates were < 70% and < 30% after 240 min in the presence of 0.4 M and 0.5 M substrate, respectively (Additional file 1: Table S3). Similar results were reported for *Ti*PheDH from *T. intermedius* [39]. We speculate that high substrate concentration may have an irreversible adverse effect on the enzyme [40].

### Continuous substrate fed-batch l-HPA synthesis

Substrate concentration is a crucial factor in industrially feasible biocatalytic processes. Therefore, a combination of substrate fed-batch with product removal strategy was applied for continuous l-HPA synthesis. During the reaction course, 2-OPBA was repeatedly added to the initial NH_4_OH/HCOONH_4_ reaction medium (pH 8.5) containing 0.3 mM NAD^+^, 3 M NH_4_^+^, 10 g L^−1^ cell-free extracts of M3–2, and 12 g L^−1^ cell-free extracts of GluDH. The substrate concentration was fixed at ~ 0.3 M to maintain high reaction efficiency. Meanwhile, l-HPA product was continuously precipitated from the reaction solution and isolated after filtration and drying. Consequently, 1.08 M 2-OPBA with a quantitative conversion of 90.2% was effectively transformed after four times fed-batch over 210 min (Fig. 4f), and the specific space–time conversion reached 30.9 mmol g^−1^ L^−1^ h^−1^. Comparisons with previously reported biocatalysis processes revealed that our M3–2 had the highest substrate loading and specific space–time conversion in l-HPA production (Additional file 1: Table S4). Furthermore, M3–2 displayed perfect stereoselectivity and yielded l-configuration HPA with up to 99% enantiomeric excess (ee). These results underscore the strong potential of M3–2 as a biocatalyst for continuous l-HPA synthesis.

### Docking simulation and tunnel analysis of wild type and derived mutants

**Fig. 5:**
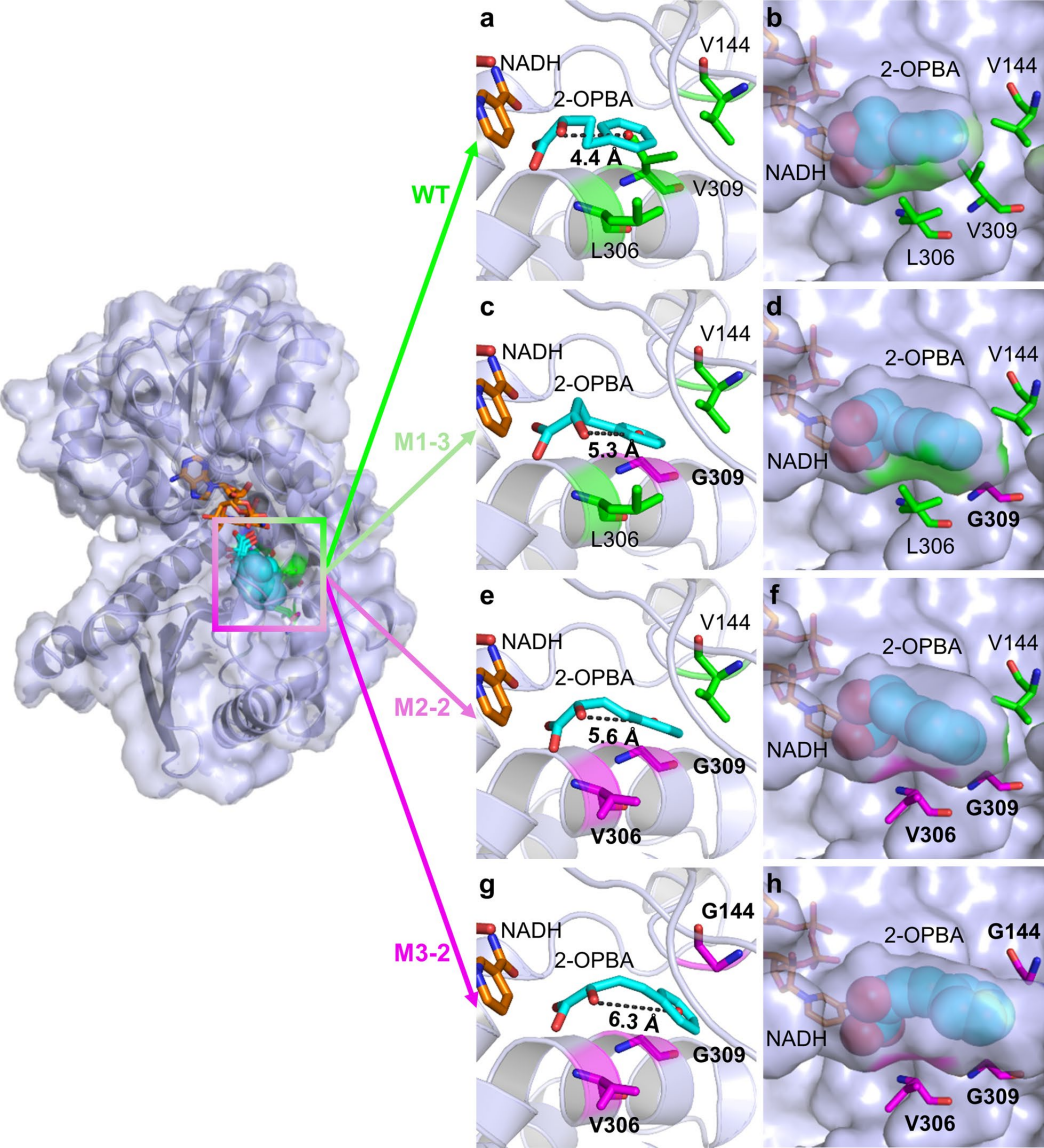
Molecular docking of 2-OPBA with *Bb*PheDH and derived mutants. Binding poses of 2-OPBA with wild-type *Bb*PheDH (**a**, **b**) and the derived mutants M1–3 (**c**, **d**), M2–2 (**e**, **f**), and M3–2 (**g**, **h**). Structural homology models of the enzyme are shown in light blue cartoon or surfaces representations. Substrate 2-OPBA is shown as cyan sticks or balls. Initial residues V144, L306, and V309 are shown as yellow sticks. Mutagenized residues G144, V306, and G309 are shown as magenta sticks. Coenzyme NADH is shown as orange sticks

To better understand the enhancement in the turnover frequency (*k*_cat_) observed in enzyme-kinetics experiments at the atomic and molecular levels, we performed docking simulations using the available structural information. The substrate 2-OPBA was docked into the catalytic active center of the wild-type *Bb*PheDH and of the derived mutants M1–3, M2–2, and M3–2 (Fig. 5). The docking simulations revealed poor adaptability of 2-OPBA to the substrate-binding pocket of wild-type *Bb*PheDH, compared with the binding pose of the native substrate PPA (Additional file 2: Figure S8). The relatively bulky hydrophobic phenyl group side chain of 2-OPBA could not fit into the substrate-binding pocket of wild-type *Bb*PheDH in a relatively stretched configuration (Fig. 5a, b), which may prevent the hydride transfer of the substrate in proper orientation. The low degree of freedom of the phenyl group side chain may be the reason for the low *k*_cat_ value, and thereby, for the low *k*_cat_/*K*_m_ value of the wild-type *Bb*PheDH toward 2-OPBA. This observation was similar to that in a previous study [41]. In contrast, the incorporation of three crucial mutations, V309G, L306V, and V144G, gradually enlarged the volume of the substrate-binding pocket compared with that of *Bb*PheDH. With this change, the bulky phenyl group side chain of 2-OPBA could fit into the enlarged substrate-binding pocket with a relatively stretched configuration at a high degree of freedom (Fig. 5c–h). Hence, the mutant M3–2 showed an enhanced *k*_cat_ value toward 2-OPBA compared with that of the wild-type *Bb*PheDH. Moreover, the observed enlargement of the substrate-binding pocket can be further supported by calculating the volume of the substrate-binding pocket, which revealed that the volume of the eventual mutant M3–2 was about 196.9 Å^3^ larger than that of the wild-type *Bb*PheDH (Additional file 1: Table S5). These results demonstrated that the enlargement of the substrate-binding pocket in a reasonable range may be beneficial to enhance the *k*_cat_ value of enzymes toward the substrates with bulky hydrophobic side chain [32].

**Fig. 6:**
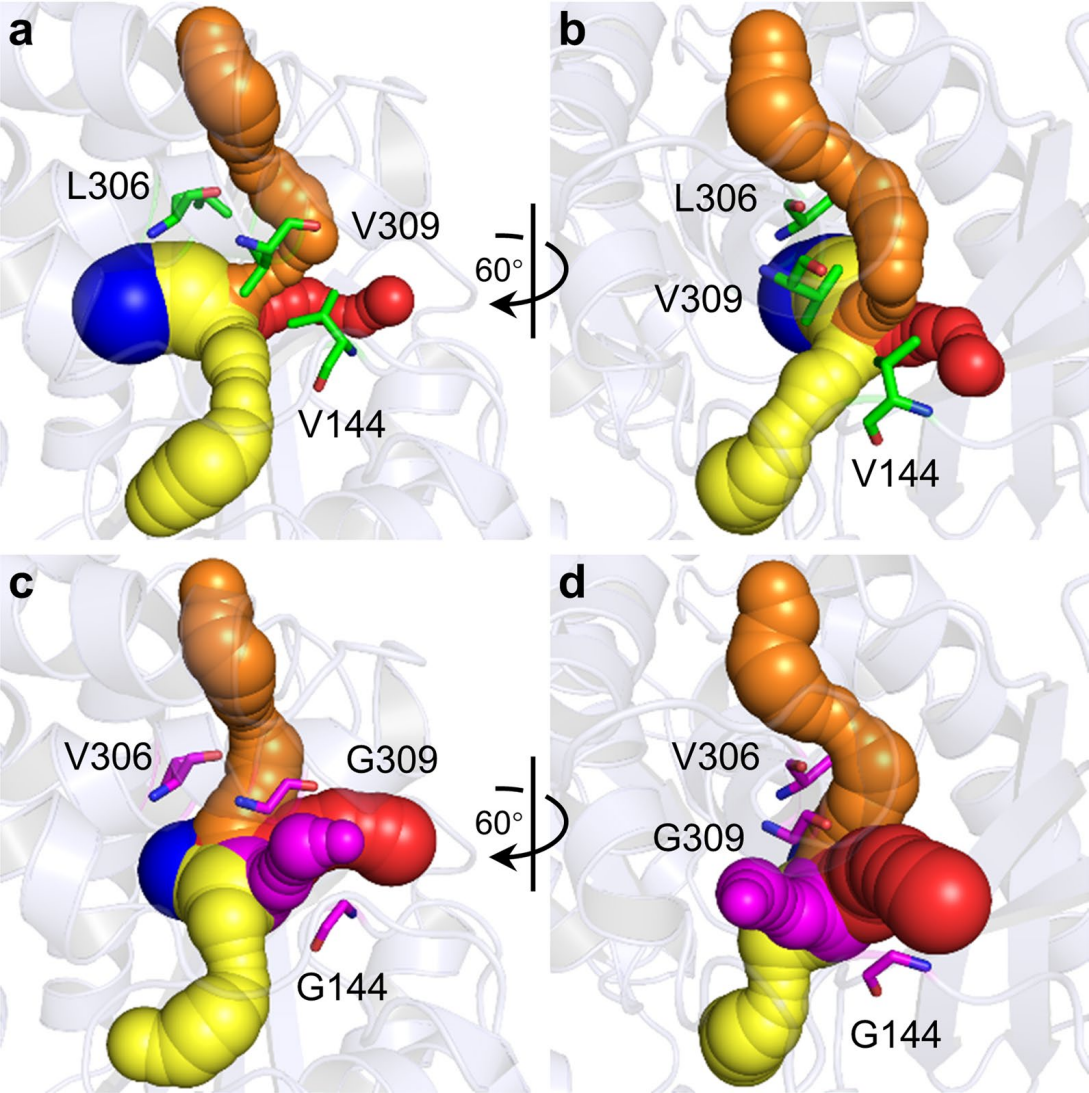
Tunnels of wild-type *Bb*PheDH (**a**, **b**) and optimal mutant M3–2 (**c**, **d**) calculated by CAVER plugin of PyMOL. Structural homology models of the enzyme are shown in light blue cartoon. Calculated tunnels are shown as balls. Initial residues V144, L306, and V309 are shown as yellow sticks. Mutagenized residues G144, V306, and G309 are shown as magenta sticks

Tunnels represent potential transport pathways for small molecules, water molecules, and ions, and play a significant role in the functioning of a large variety of proteins [42, 43]. The accessibility of a tunnel depends largely on its shape, size, and amino acid composition. To a certain extent, it can be modified by protein engineering [44, 45]. Therefore, we investigated whether steric hindrance engineering alters *Bb*PheDH tunnels. Tunnels analyses based on the structure homology model of M3–2 revealed that, compared with *Bb*PheDH, the incorporation of V144G, L306V, and V309G formed a new tunnel (shown in magenta) in the vicinity of the catalytic active center (Fig. 6), suggesting a new transport pathway for substrate access and product egress. Furthermore, V144G, L306V, and V309G mutations markedly altered the shapes of the existing tunnels (Fig. 6). The widened tunnels (shown in red and orange) could further accelerate the transport rates of substrate and product. Both pieces of information lend support to the enhancement of the *k*_cat_ value toward 2-OPBA catalyzed by M3–2 observed in the enzyme-kinetics experiments. This observation demonstrated that the formation of new tunnels and widening of existing ones can modulate the catalytic activity of enzymes. It also resembled the findings reported for prior studies [46, 47].

## Conclusions

Here, we employed a modified steric hindrance engineering approach to create an enhanced biocatalyst with *Bb*PheDH as a mutagenesis template for efficient l-HPA synthesis. Seven superior mutants were screened from the mutagenesis libraries constructed based on molecular docking, double-proximity filtering, and a degenerate codon. The *k*_cat_ and *k*_cat_/*K*_m_ values of the mutant M3–2 (V309G/L306V/V144G) were 12.7- and 12.9-fold higher than those of the wild type, respectively, it converted 1.08 M 2-OPBA to l-HPA in 210 min, and its specific space–time conversion was 30.9 mmol g^−1^ L^−1^ h^−1^. The substrate loading and specific space–time conversion are the highest values reported so far, demonstrating that mutant M3–2 is a useful biocatalyst with potential for the industrial production of l-HPA. Structure analysis demonstrated that mutations altered the substrate-binding pocket and tunnel, and variations in steric hindrance modulate the catalytic activity of *Bb*PheDH towards 2-OPBA. Moreover, our study indicates the combination of molecular docking with a double-proximity filter as a promising workflow for capturing the proximal steric hindrance synergistic effects. The modified steric hindrance engineering approach can be a valuable addition and extension of the current enzyme engineering toolbox.

## Methods

### Strains, plasmids, and chemicals

PheDHs from *Bacillus badius* (*Bb*PheDH) and *Geobacillus kaustophilus* (GkPheDH) were generated in the laboratory. Glucose dehydrogenase from *Bacillus amyloliquefaciens* (*Ba*GluDH) was purchased from Sigma-Aldrich Corp. (Beijing, China). *Escherichia coli* BL21 (DE3) and plasmid pET-28a (+) were procured from Novagen (Nanjing, China) and used as the gene expression host and vector, respectively. The 2-oxo-4-phenylbutyric acid (2-OPBA) and l-homophenylalanine (l-HPA) were acquired from J&K Chemical (Beijing, China). Isopropyl-β-d-thiogalactopyranoside (IPTG), kanamycin, NADH, and NAD^+^ were obtained from TCI (Shanghai, China). All other chemicals were purchased from commercial sources, at least reagent grade, and used without further purification.

### Sequence alignment and site-directed mutagenesis

Amino acid sequence alignment was performed using the MUSCLE server (https://www.ebi.ac.uk/Tools/msa/muscle/) [48] and displayed using the Esprit server (http://espript.ibcp.fr) [49]. Site-directed mutagenesis was introduced by whole-plasmid PCR [38] using primers containing the required degenerate codons and the pET-28a (+) plasmid inserts the *pdh* gene as the template. The PCR products were analyzed by agarose gel electrophoresis and transformed into *E. coli* BL21 (DE3) cells. All target mutants were isolated from the constructed mixed libraries and confirmed by DNA sequencing (Talen-Bio, Wuxi, China).

### Protein expression and purification

*Escherichia coli* BL21 (DE3) cells containing recombinant pET-28a (+) plasmids were cultured in Luria–Bertani (LB) medium with kanamycin (50 mg L^−1^) at 37 °C and with shaking at 200 rpm for 2 h. Enzyme expression was induced with IPTG (final concentration = 0.2 mM) when the bacteria reached OD_600_ = 0.6–0.8. The cells were incubated at 20 °C and with shaking at 200 rpm for 12 h. *His*-tagged enzymes were purified with an ÄKTA purifier system (GE Healthcare, Little Chalfont, UK). The cells were resuspended in buffer A (100 mM potassium phosphate, 150 mM NaCl, and 20 mM imidazole; pH 7.5) and disrupted with an ultrasonic cell disruption system (SCIENTZ-IID; Ningbo Scientz Biotechnology, Ningbo, Zhejiang, China) followed by centrifugation at 12,000 rpm and 4 °C for 30 min to remove cell debris and obtain cell-free extracts. The latter were passed through a 0.22-μm filter and loaded into a standard Ni–NTA affinity column (Thermo Fisher Scientific, Waltham, MA, USA) pre-equilibrated with buffer A. The column was then gradient-eluted with buffer B (100 mM potassium phosphate, 150 mM NaCl, and 500 mM imidazole; pH 7.5). Eluted fraction purity was determined by SDS-PAGE (Additional file 2: Figure S9).

### Activity and kinetic parameters analysis

Reductive amination activity was determined at a 200-μL scale in 96-well microtiter plates at 30 °C. The rate of initial decrease of the absorbance was measured at 340 nm (indicating NADH consumption) using a MultiSkan GO UV-spectrometer (Thermo Fisher Scientific). The reaction mixture consisted of NH_4_Cl/NH_4_OH buffer (2 M; pH 9.5), 10 mM 2-OPBA, 0.2 mM NADH, and a certain amount of cell-free extract. One unit of enzyme activity (1 U) was defined as the amount of enzyme oxidizing 1 μM NADH per minute. Kinetic parameters with respect to 2-OPBA (PPA) were evaluated in NH_4_Cl/NH_4_OH buffer (2 M; pH 9.5) at 30 °C in the presence of 1–20 mM 2-OPBA (PPA) and 0.5 mM NADH with the purified enzymes. Kinetic parameters with respect to NADH were evaluated in NH_4_Cl/NH_4_OH buffer (2 M; pH 9.5) at 30 °C in the presence of 0.1–0.5 mM NADH and 20 mM 2-OPBA with the purified enzymes (Additional file 1: Table S6). *K*_m_ and *V*_max_ were calculated in GraphPad Prism 8 (GraphPad Software, La Jolla, CA, USA) by nonlinear curve fitting of the initial rate versus substrate concentration data to the Michaelis–Menten equation.

### Thermostability analysis

Thermostability assays of wild-type *Bb*PheDH and its mutant were conducted by measuring *T*_50_^30^, which is the temperature at which 50% of the enzyme activity is lost after 30 min heat treatment. The enzymes were incubated at various temperatures (30–70 °C) for 30 min, and their residual activity was measured. Specific activity before incubation was normalized as 100%. Enzyme activity was measured in NH_4_Cl/NH_4_OH buffer (2 M; pH 9.5) containing 0.2 mM NADH and 10 mM 2-OPBA.

### Asymmetric reductive amination of 2-OPBA to l-HPA

PheDH was coupled with GluDH for the asymmetric reductive amination of 2-OPBA. The total volume of the biocatalytic reaction mixture was 5 mL. It contained cell-free extracts of *E. coli* wet cells (10 g L^−1^ PheDH and 12 g L^−1^ GluDH), 2-OPBA (0.1–0.5 M), glucose (0.12–0.6 M), NAD^+^ (0.05–1.0 mM), and NH_4_OH/HCOONH_4_ buffer (1–5 M; pH 7.5–9.5). The reaction mixture was incubated at 25–50 °C with shaking at 200 rpm. Samples (200 μL) were drawn at intervals and alkalized with 200 μL NaOH (10 M) to terminate the reaction. The solutions were then passed through a 0.22 μm filter and subjected to High-Performance Liquid Chromatography (HPLC).

### Fed-batch synthesis of l-HPA

The initial reaction mixture (100 mL) consisted of cell-free extract (10 g L^−1^ PheDH and 12 g L^−1^ GluDH), 2-OPBA (0.3 M), glucose (0.36 M), NAD^+^ (0.3 mM), and NH_4_OH/HCOONH_4_ buffer (3 M; pH 8.5). The reaction was initiated by adding PheDH and GluDH and incubated at 30 °C with shaking at 200 rpm. The pH value was maintained at 8.5 by titrating concentrated NH_3_·H_2_O during the reaction. Over the 30–75 min reaction period, the precipitated l-HPA was separated by filtration. The filtrate contained enzymes and cofactors and was used as the reaction medium for the next reaction period. The latter was initiated by adding 0.3 M 2-OPBA and 0.36 M glucose. Four fed-batch cycles were performed, and the 2-OPBA conversion and l-HPA enantiomeric excess (ee) values were determined by HPLC.

### HPLC analysis

The conversion of 2-OPBA and the ee value of l-HPA were determined with an Agilent 1260 HPLC (Agilent Technologies, Santa Clara, CA, USA). The absolute l-HPA configuration was established by comparing it with authentic reference material after derivatization. The conditions for determining the 2-OPBA conversion rate were as follows: Diamonsil C18(2) column (5 μm; 250 mm × 4.6 mm) (Phenomenex, Torrance, CA, USA); injection volume, 10 μL; mobile phase A [55% (v/v) methanol plus 0.1% (v/v) trifluoroacetic acid], 20 min at 30 °C; flow rate, 0.8 mL min^−1^; and UV detection at 230 nm. The 2-OPBA retention time was 9.04 min. The ee value of l-HPA was determined using a Develosil® ODS-UG-5 column (5 μm; 150 mm × 4.6 mm) (Phenomenex) after derivatization with 1-fluoro-2,4-dinitrophenyl-5-l-alanineamide (FDAA). A 5-μL reaction sample, 4 μL of 1 M NaHCO_3_, and 20 μL of 1% (w/v) FDAA in acetone were mixed and heated at 40 °C for 60 min. Then 4 μL of 1 M HCl and 467 μL of 40% (v/v) aqueous acetonitrile were added to the mixture. The latter was then passed through a 0.22-μm filter and subjected to HPLC. The conditions for determining the ee value of l-HPA were as follows: injection volume, 20 μL; mobile phase B (5% (v/v) acetonitrile, 0.05% (v/v) trifluoroacetic acid, and 1% (v/v) methanol); mobile phase C (60% (v/v) acetonitrile, 0.05% (v/v) trifluoroacetic acid, and 1% (v/v) methanol); linear gradient from 0 to 100%; mobile phase C, 45 min at 30 °C; flow rate, 1.0 mL min^−1^; and UV detection at 340 nm. The l-HPA retention time was 33.65 min.

### Homology modeling and molecular docking

Structural homology models of *Bb*PheDH and its mutants were constructed using the SWISS-MODEL server (https://swissmodel.expasy.org/) [50] based on the crystal structure of the PheDH from *Rhodococcus* sp. M4 (PDB: 1C1D) [34]. The 3D structure of the ligand was obtained from the PubChem website (https://pubchem.ncbi.nlm.nih.gov/) [51]. Ligand energy was minimized with the OPTIMIZE plugin of PyMOL [52]. AutoDock v. 4.2 [53] was used for docking simulations. Prior to docking, each protein was protonated at pH 9.5 using the H++ server (http://biophysics.cs.vt.edu/H++) [54] to mimic the experimental condition. All water molecules were removed, and nonpolar hydrogen atoms were added with MGLTools v. 1.5.4. The components of the catalytic triad (K78, K90, and N276) were defined as flexible residues. A grid box of 50 × 50 × 50 Å with 0.375 Å spacing encompassed the active cavities of *Bb*PheDH and its mutants and was set as the search space for suitable substrate-binding poses. Energies of the interactions between the ligands and the receptors were calculated with MGLTools v. 1.5.4. The lowest energy docking pose was selected for the subsequent molecular dynamics simulations.

### Tunnel analysis

The tunnels of *Bb*PheDH and its mutant M3–2 were calculated with the CAVER v. 3.0 plugin of PyMOL [55]. The probe radius was set to 1.2 Å, the default settings were applied, and the center of the catalytic triad (K78, K90, D125, and N276) was the starting point. All water molecules were removed, nonpolar hydrogen atoms were added with MGLTools v. 1.5.4, and the volumes of the substrate-binding pockets of *Bb*PheDH and its mutants were calculated with the ProteinsPlus tool (https://proteins.plus/) [56]. The PDB format files were uploaded for the calculations.

## Supplementary Information

**Additional file 1: Table S1.** Primers used for site-directed mutagenesis*.* **Table S2.** Enzyme thermostabilities of *Bb*PheDH and its superior mutants. **Table S3.** Effects of substrate concentration on the asymmetric reductive amination of 2-OPBA catalyzed by mutant M3–2. **Table S4.** Comparison between M3–2 and other reported PheDHs for the synthesis of l-HPA from 2-OPBA. **Table S5.** Calculated substrate-binding pocket volume and area of *Bb*PheDH and its superior mutants. **Table S6.** Kinetic parameters of *Bb*PheDH and its superior mutants toward NADH.

**Additional file 2: Figure S1.** Time course of asymmetric reductive amination of 2-OPBA catalyzed by *Bb*PheDH and *Gk*PheDH. **Figure S2.** Amino acid sequence alignment of the PheDHs from different sources*.* **Figure S3.** Relative activity of the single-site mutants constructed in the first round of steric hindrance engineering. **Figure S4.** Relative activity of the double-site mutants constructed in the second round of steric hindrance engineering. **Figure S5.** Relative activity of the triple-site mutants constructed in the third round of steric hindrance engineering. **Figure S6.** Relative activity of the quadruple-site mutants constructed in the fourth round of steric hindrance engineering. **Figure S7.** Relative activity of *Bb*PheDH and GluDH in different concentrations of NH_4_OH/HCOONH_4_ buffer (pH 8.5). **Figure S8.** Binding poses of native substrate PPA (**a**) and bulky substrate 2-OPBA (**b**) with *Bb*PheDH. **Figure S9.** SDS-PAGE analysis of *Bb*PheDH and its superior mutants.

## Data Availability

All data generated or analyzed during this study are included in this published article and its Additional files.

## Abbreviations

l-HPA: l-Homophenylalanine
2-OPBA: 2-Oxo-4-phenylbutyric acid
PheDH: Phenylalanine dehydrogenase
PPA: Phenylpyruvic acid
GluDH: Glucose dehydrogenase
HPLC: High-Performance Liquid Chromatography
