# Supplementary material for: Structure-guided steric hindrance engineering of Bacillus badius phenylalanine dehydrogenase for efficient l-homophenylalanine synthesis
Source: Biotechnol Biofuels. 2021 Oct 24;14:207. doi: 10.1186/s13068-021-02055-0 (PMC8543943; doi:10.1186/s13068-021-02055-0)
Supplement: Supplementary file 1 — Additional file 1: Table S1. Primers used for site-directed mutagenesis. Table S2. Enzyme thermostabilities of BbPheDH and its superior mutants. Table S3. Effects of substrate concentration on the asymmetric reductive amination of 2-OPBA catalyzed by mutant M3–2. Table S4. Comparison between M3–2 and other reported PheDHs for the synthesis of l-HPA from 2-OPBA. Table S5. Calculated substrate-binding pocket volume and area of BbPheDH and its superior mutants. Table S6. Kinetic parameters of BbPheDH and its superior mutants toward NADH. [file 13068_2021_2055_MOESM1_ESM.docx]

# Additional file 1

**Table S1. Primers used for site-directed mutagenesis**

| Round | Primer | Sequence (5’-3’) |
| --- | --- | --- |
| 1 | L50-R | GGCCCAGCAGBNGGAGGATGTCGCATGCAGCC |
|  | L50-F | CGACATCCTCCNVCTGCTGGGCCTAAAGTGGT |
|  | C53-R | GCACTTGGAGGAGSNCGCATGCAGCCTTACAATAG |
|  | C53-F | GGCTGCATGCGNSCTCCTCCAAGTGCTGG |
|  | M75-R | GCTTGTCGAAAGGCDBNACATATAAGTGTG |
|  | M75-F | CTTATATGTNVHGCCTTTCGACAAGCGCAACGC |
|  | T122-R | GGACGTTTTTACGSNGGCACAGATATGGGG |
|  | T122-F | CTGTGCCNSCGTAAAAACGTCCTCCAAGGCTATC |
|  | T124-R | CACGGGCGSNGATATGGGGACCAATATGGAAG |
|  | T124-F | GGTCCCCATATCNSCGCCCGTGTAAAAACG |
|  | V144-R | CGAATTGCATCGSNGGAGTACCGGAGGCTTATGG |
|  | V144-F | CCGGTACTCCNSCGATGCAATTCGTCTCCTTC |
|  | S156-R | GGCGACGSNTCTATTCCGACTGCCATGG |
|  | S156-F | CGGAATAGANSCGTCGCCTCCACCTCCAT |
|  | L306-R | GTTAATTCAGGAGGCGBNATCCAAGTAGCTGACG |
|  | L306-F | GCTACTTGGATNVCGCCTCCTGAATTAACAATG |
|  | V309-R | GGCCTTATCCAAGSNGCTGACGAATTGTATG |
|  | V309-F | CGTCAGCNSCTTGGATAAGGCCTCCTGAATTAAC |
| 2 | L50-R | GGCCCAGCAGBNGGAGGATGTCGCATGCAGCC |
|  | L50-F | CGACATCCTCCNVCTGCTGGGCCTAAAGTGGT |
|  | V144-R | CGAATTGCATCGSNGGAGTACCGGAGGCTTATGG |
|  | V144-F | CCGGTACTCCNSCGATGCAATTCGTCTCCTTC |
|  | S156-R | GGCGACGSNTCTATTCCGACTGCCATGG |
|  | S156-F | CGGAATAGANSCGTCGCCTCCACCTCCAT |
|  | L306-2-R | GTTAATTCAGGAGGCGBNATCCAAGGTGCTGACG |
|  | L306-2-F | GCACCTTGGATNVCGCCTCCTGAATTAACAATG |
| 3 | L50-R | GGCCCAGCAGBNGGAGGATGTCGCATGCAGCC |
|  | L50-F | CGACATCCTCCNVCTGCTGGGCCTAAAGTGGT |
|  | M75-R | GCTTGTCGAAAGGCDBNACATATAAGTGTG |
|  | M75-F | CTTATATGTNVHGCCTTTCGACAAGCGCAACGC |
|  | V144-R | CGAATTGCATCGSNGGAGTACCGGAGGCTTATGG |
|  | V144-F | CCGGTACTCCNSCGATGCAATTCGTCTCCTTC |
| 4 | T122-R | GGACGTTTTTACGSNGGCACAGATATGGGG |
|  | T122-F | CTGTGCCNSCGTAAAAACGTCCTCCAAGGCTATC |
|  | T124-R | CACGGGCGSNGATATGGGGACCAATATGGAAG |
|  | T124-F | GGTCCCCATATCNSCGCCCGTGTAAAAACG |

**Table S2. Enzyme thermostabilities of *Bb*PheDH and its superior mutants**

| Enzyme | *T*_50_^30^*^a^* (°C) |
| --- | --- |
| *Bb*PheDH | 62 |
| M1-1 | 60 |
| M1-2 | 59 |
| M1-3 | 54 |
| M2-1 | 60 |
| M2-2 | 58 |
| M3-1 | 63 |
| M3-2 | 59 |

*^a^T*_50_^30^ value: the temperature at which 50% of enzyme activity is lost following a heat treatment for 30 min. The enzyme was incubated at various temperatures (30-70 °C) for 30 min, and their residual activity was detected. Specific activity before incubation was normalized as 100%. Activity was measured in NH_4_Cl/NH_4_OH buffer (2 M, pH 9.5) containing 10 mM 2-OPBA and 0.2 mM NADH at 30 ℃ and carried out at a 200-μL scale in 96-well microtiter plates by monitoring the initial decrease velocity of the absorbance at 340 nm (indicating NADH consumption).

**Table S3. Effects of substrate concentration on the asymmetric reductive amination of 2-OPBA catalyzed by mutant M3-2**

| Substrate concentration (mM) | 200 | 300 | 400 | 500 |
| --- | --- | --- | --- | --- |
| Conversion (%)*^a^* | 99.9 | 99.9 | 59.2 | 20.5 |
| Initial reaction velocity (mM·min^-1^) | 15.6 | 16.8 | 8.1 | 2.2 |
| Inhibition (%) | - | - | 51.8 | 86.9 |

*^a^*Conversion was determined by monitoring the concentration of 2-OPBA in the reaction mixture with HPLC analysis at 2 h.

**Table S4. Comparison between M3-2 and other reported PheDHs for the synthesis of L-HPA from 2-OPBA**

| Biocatalyst | 2-OPBA  (mM) | Cell loading*^a^*  (g·L^−1^) | Reaction  time (h) | Specific space-time conversion  (mmol·g^−1^·L^−1^·h^−1^) |
| --- | --- | --- | --- | --- |
| PheDH/GDH | 100 | 10 | 2 | 5.0 |
| *Bb*PheDH-M3-2/GDH | 300 | 10 | 0.5 | 60.0 |
| *Bb*PheDH-M3-2/GDH | 1080 | 10 | 3.5 | 30.9 |
| *Rs*PheDH/FDH | 60 | - | - | - |
| *Rs*PheDH/FDH | 67*^b^* | - | - | - |
| *Ti*PheDH/FDH | 100*^c^* | 5.4*^d^* | 2 | 9.3 |
| *Ti*PheDH/FDH | 510 | 5.4*^d^* | 24 | 3.9 |

*^a^*Cell loading: Cell-free extracts of *E. coli* cells (PheDHs). *^b^*The 2-OPBA loading was converted from the data of the mass fraction (1.2% w/v). *^c^*The concentration of 2-OPBA was 20 g·L^−1^. *^d^*The cell loading was converted from the data of the specific activity (1.3 U·mg^−1^ of cell-free extracts) of the *Ti*PheDH and its concentration (7 U·mL^−1^).

**Table S5. Calculated substrate-binding pocket volume and area of *Bb*PheDH and its superior mutants**

| Enzyme | Volume (A^3^) | Area (A^2^) |
| --- | --- | --- |
| *Bb*PheDH | 529.3 | 691.9 |
| M1-3 | 611.4 | 799.2 |
| M2-2 | 651.0 | 851.2 |
| M3-2 | 726.2 | 942.9 |

**Table S6. Kinetic parameters of *Bb*PheDH and its superior mutants toward NADH**

| Enzyme | *k*_cat_ (s^-1^) | *K*_m_ (μM) | *k*_cat_/*K*_m_ (mM^-1^·s^-1^) |
| --- | --- | --- | --- |
| *Bb*PheDH | 8 ± 1 | 65 ± 3 | 115 ± 14 |
| M1-1 | 22 ± 1 | 141 ± 7 | 157 ± 1 |
| M1-2 | 43 ± 2 | 98 ± 4 | 446 ± 4 |
| M1-3 | 71 ± 3 | 111 ± 5 | 635 ± 3 |
| M2-1 | 72 ± 4 | 75 ± 2 | 962 ± 40 |
| M2-2 | 96 ± 4 | 90 ± 3 | 1069 ± 13 |
| M3-1 | 92 ± 4 | 85 ± 3 | 1096 ± 13 |
| M3-2 | 83 ± 4 | 74 ± 2 | 1076 ± 34 |

Activity was measured in NH_4_Cl/NH_4_OH buffer (2 M, pH 9.5) containing 0.1-0.5 mM NADH and 20 mM 2-OPBA at 30 ℃ and carried out at a 200-μL scale in 96-well microtiter plates by monitoring the initial decrease velocity of the absorbance at 340 nm (indicating NADH consumption). The kinetic parameter was performed in triplicate, and error bounds represent ± sd.
