# Supplementary material for: Structure-guided steric hindrance engineering of Bacillus badius phenylalanine dehydrogenase for efficient l-homophenylalanine synthesis
Source: Biotechnol Biofuels. 2021 Oct 24;14:207. doi: 10.1186/s13068-021-02055-0 (PMC8543943; doi:10.1186/s13068-021-02055-0)
Supplement: Supplementary file 2 — Additional file 2: Figure S1. Time course of asymmetric reductive amination of 2-OPBA catalyzed by BbPheDH and GkPheDH. Figure S2. Amino acid sequence alignment of the PheDHs from different sources. Figure S3. Relative activity of the single-site mutants constructed in the first round of steric hindrance engineering. Figure S4. Relative activity of the double-site mutants constructed in the second round of steric hindrance engineering. Figure S5. Relative activity of the triple-site mutants constructed in the third round of steric hindrance engineering. Figure S6. Relative activity of the quadruple-site mutants constructed in the fourth round of steric hindrance engineering. Figure S7. Relative activity of BbPheDH and GluDH in different concentrations of NH4OH/HCOONH4 buffer (pH 8.5). Figure S8. Binding poses of native substrate PPA (a) and bulky substrate 2-OPBA (b) with BbPheDH. Figure S9. SDS-PAGE analysis of BbPheDH and its superior mutants. [file 13068_2021_2055_MOESM2_ESM.docx]

# Additional file 2


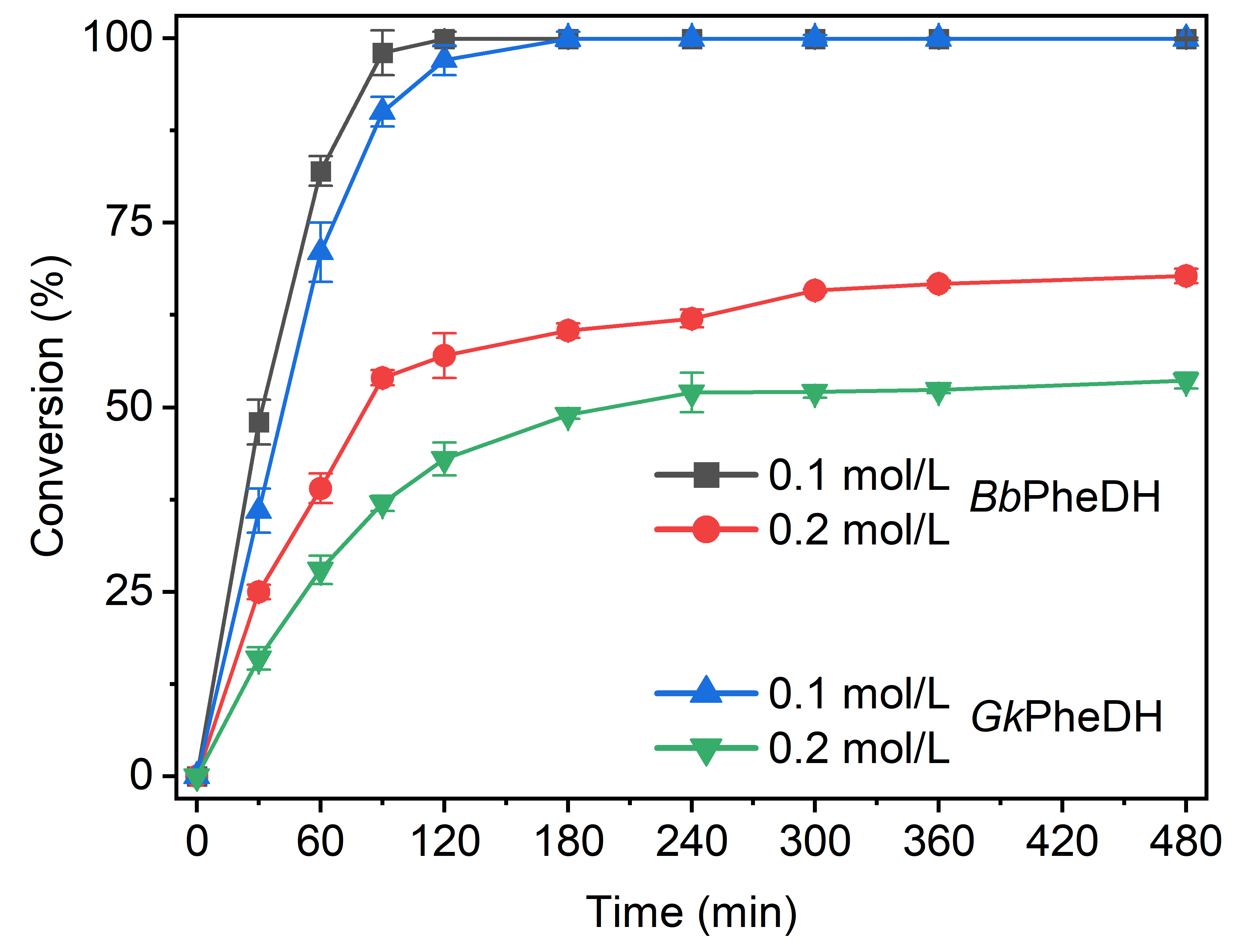


**Figure S1**. **Time course of asymmetric reductive amination of 2-OPBA catalyzed by *Bb*PheDH and *Gk*PheDH.** Reaction conditions: 0.1 M (0.2 M) 2-OPBA, 0.12 M (0.24 M) glucose, 0.5 mM NAD^+^, 1 M NH_4_OH/HCOONH_4_ buffer (pH 9.0), 10 g·L^−1^ cell-free extracts of PheDH, and 12 g·L^-1^ cell-free extracts of GluDH in a total volume of 5 mL at 30 °C and 200 rpm. The conversion was determined by monitoring the concentration of 2-OPBA with HPLC analysis. The biotransformation experiment was performed in triplicate, and error bounds represent ± sd.

**
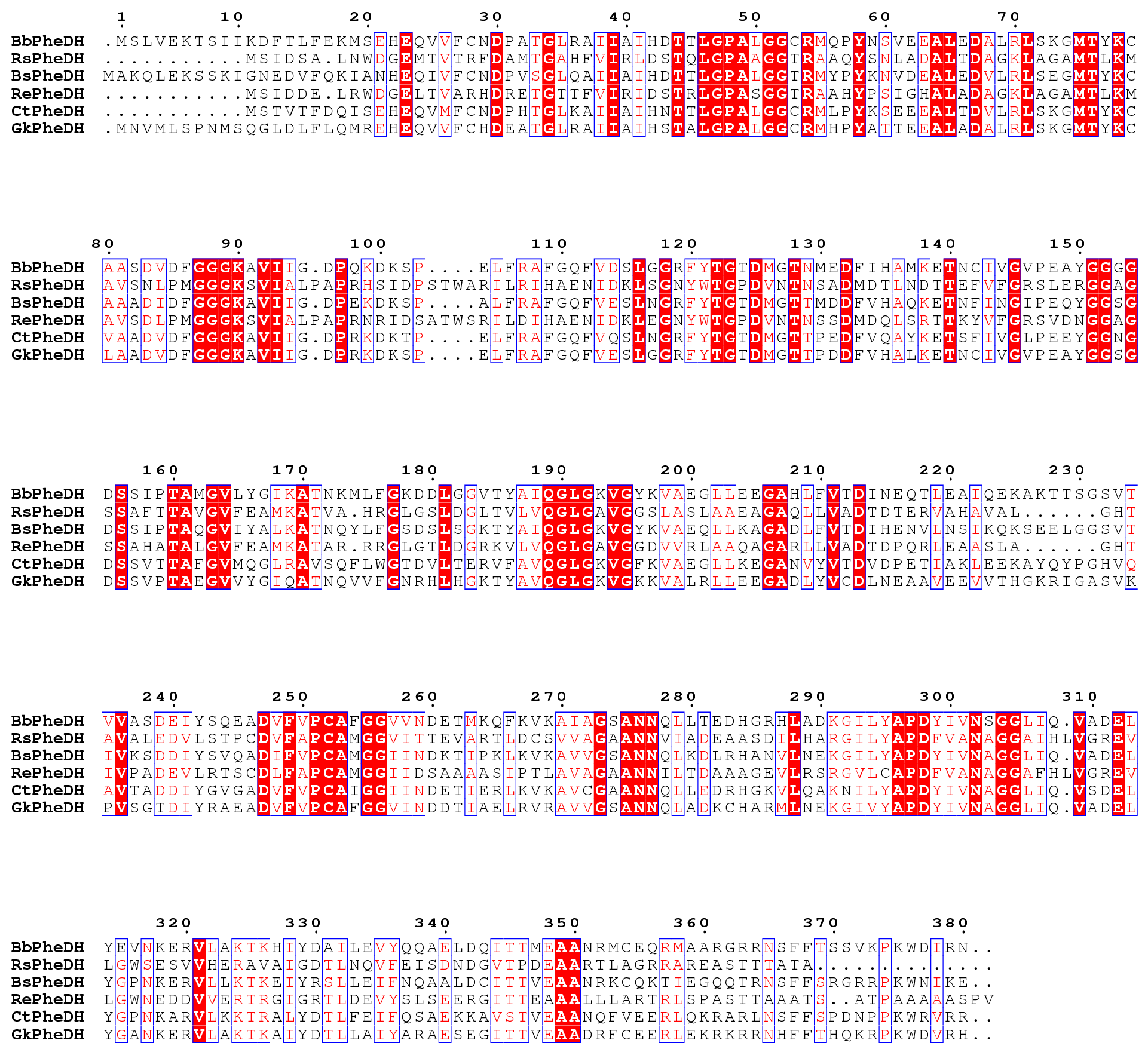
**

**Figure S2**. **Amino acid sequence alignment of the PheDHs from different sources*.*** Four conservative catalytic residues (K78, K90, D125, and N276, residue number of *Bb*PheDH) are marked with black triangles. Sequence alignment was performed using the MUSCLE server (https://www.ebi.ac.uk/Tools/msa/muscle/) and displayed using Esprit server (http://espript.ibcp.fr).

**
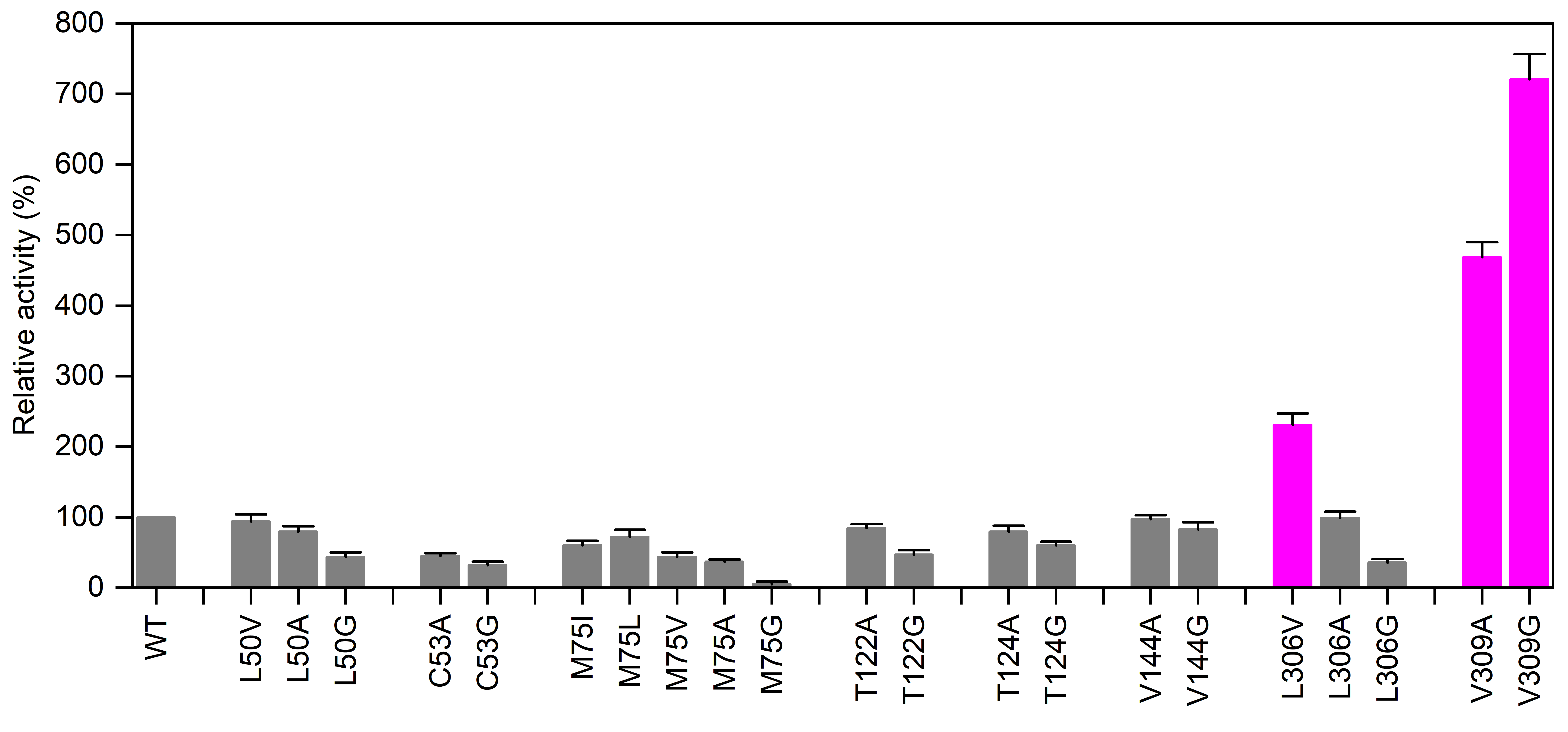
**

**Figure S3. Relative activity of the single-site** **mutants constructed in the first round of steric hindrance engineering.** Activity was carried out at a 200-μL scale in 96-well microtiter plates by monitoring the initial decrease velocity of the absorbance at 340 nm (indicating NADH consumption). The specific activity performed with wild-type *Bb*PheDH was normalized as 100%. The activity determination was performed in triplicate, and error bounds represent ± sd.

**
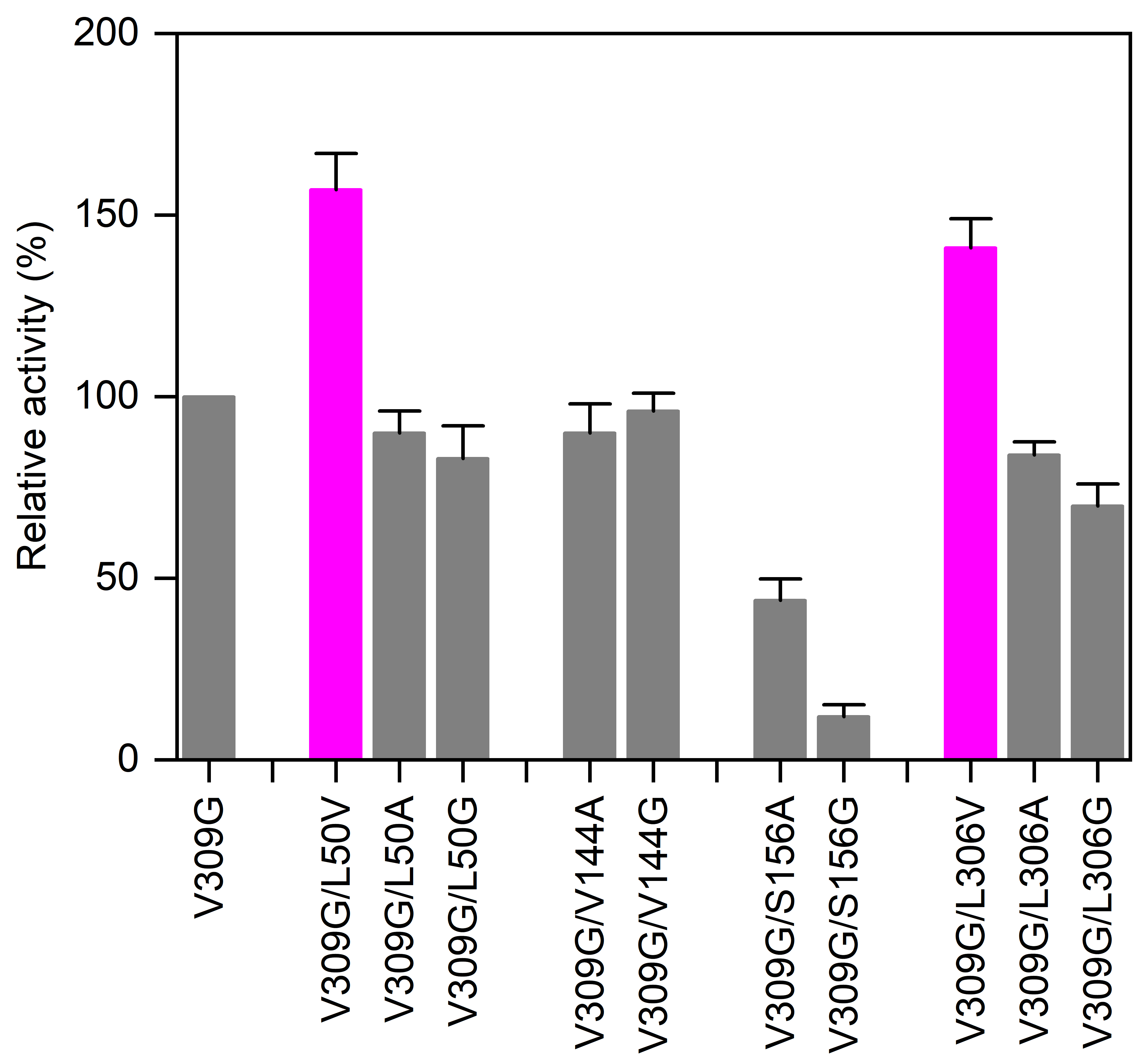
**

**Figure S4. Relative activity of the double-site mutants constructed in the second round of steric hindrance engineering.** Activity was carried out at a 200-μL scale in 96-well microtiter plates by monitoring the initial decrease velocity of the absorbance at 340 nm (indicating NADH consumption). The specific activity performed with mutant V309G was normalized as 100%. The activity determination was performed in triplicate, and error bounds represent ± sd.

**
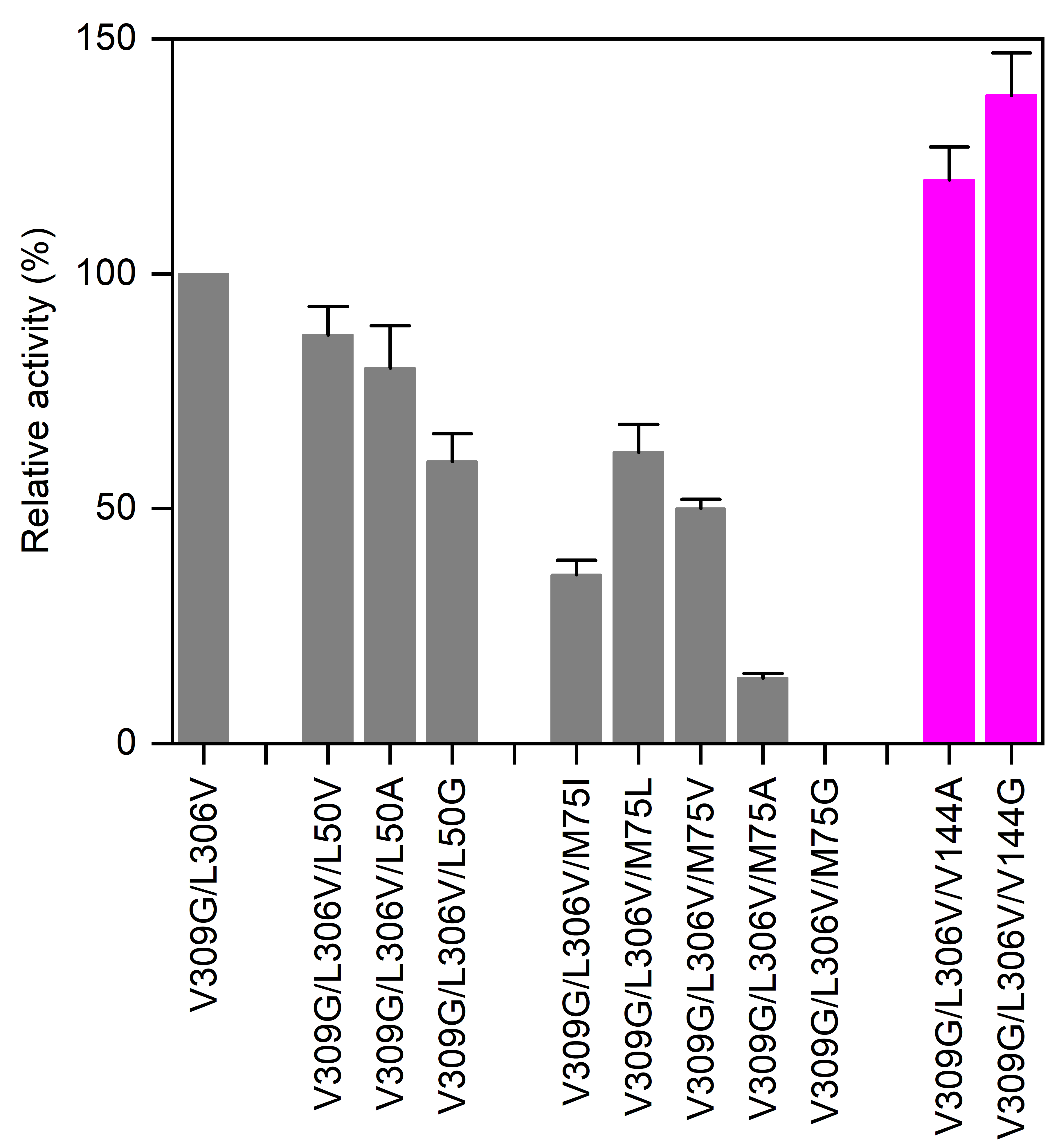
**

**Figure S5. Relative activity of the triple-site mutants constructed in the third round of steric hindrance engineering.** Activity was carried out at a 200-μL scale in 96-well microtiter plates by monitoring the initial decrease velocity of the absorbance at 340 nm (indicating NADH consumption). The specific activity performed with mutant V309G/L306V was normalized as 100%. The activity determination was performed in triplicate, and error bounds represent ± sd.

**
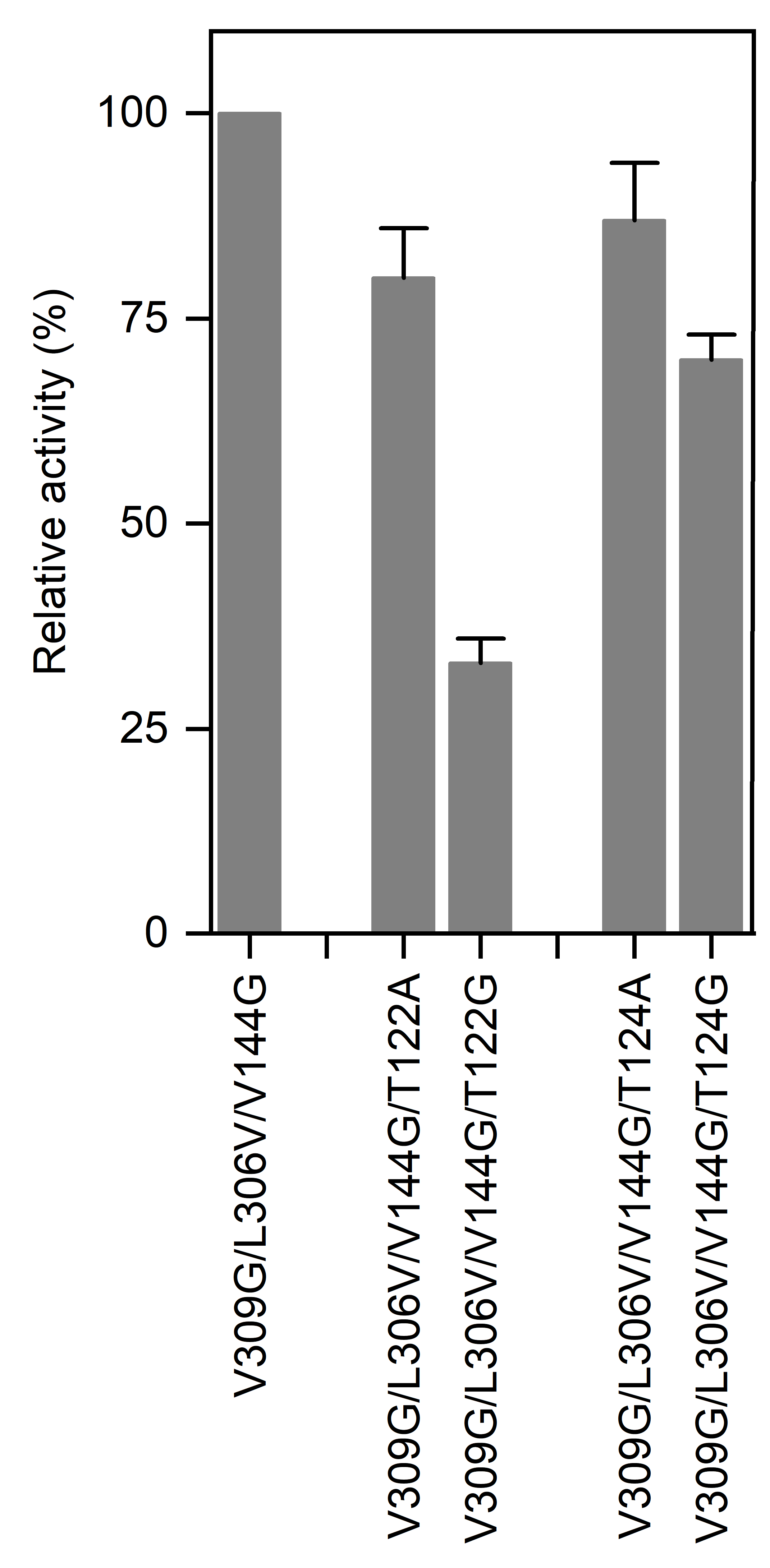
**

**Figure S6. Relative activity of the quadruple-site mutants constructed in the fourth round of steric hindrance engineering.** Activity was carried out at a 200-μL scale in 96-well microtiter plates by monitoring the initial decrease velocity of the absorbance at 340 nm (indicating NADH consumption). The specific activity performed with mutant V309G/L306V/V144G was normalized as 100%. The activity determination was performed in triplicate, and error bounds represent ± sd.

**
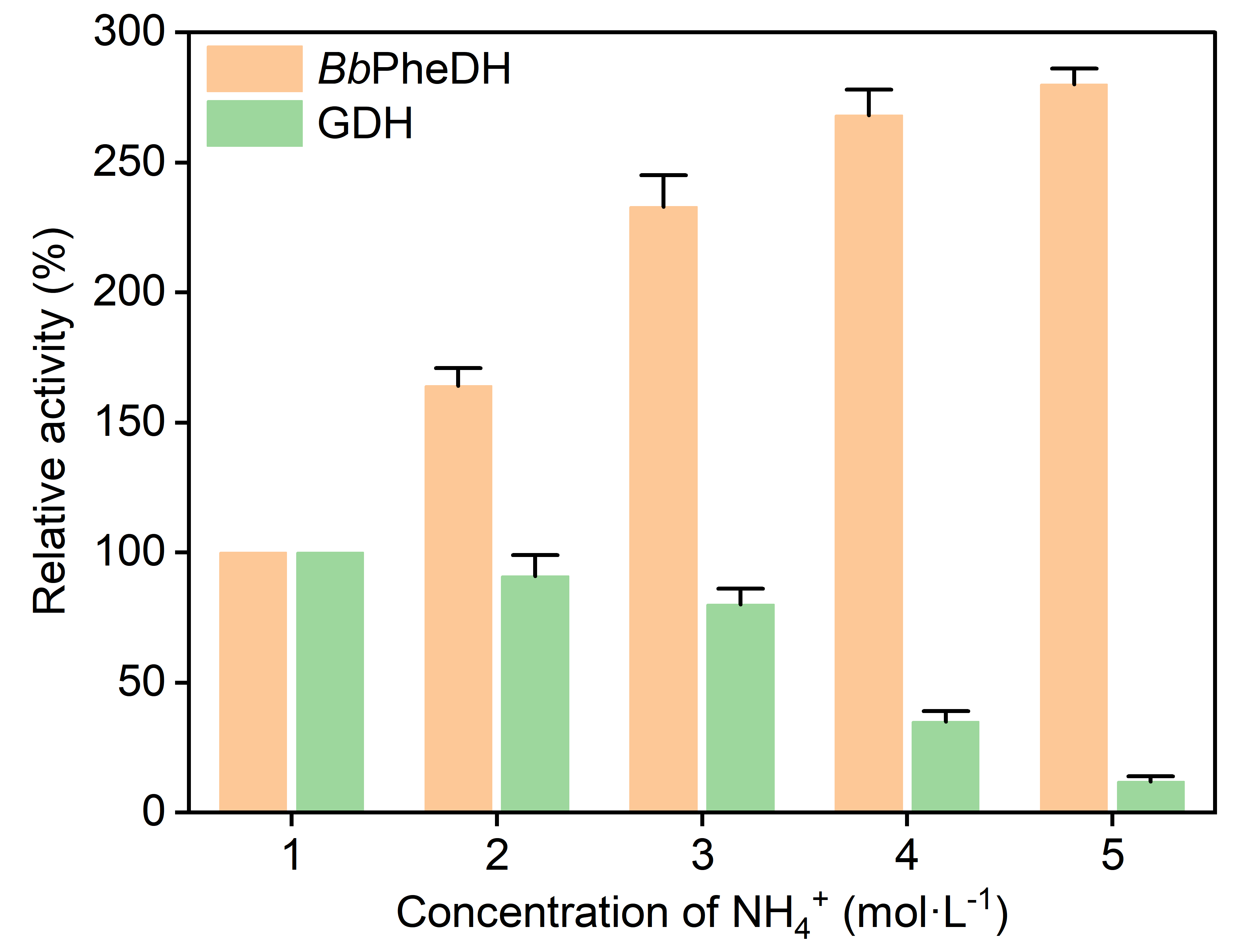
**

**Figure S7. Relative activity of *Bb*PheDH and GDH in different concentrations of** **NH_4_OH/HCOONH_4_ buffer (pH 8.5).** Activity was carried out at a 200-μL scale in 96-well microtiter plates by monitoring the initial decrease velocity of the absorbance at 340 nm (indicating NADH consumption). The specific activities of *Bb*PheDH and GDH performed in 1 M NH_4_Cl/NH_4_OH buffer (pH 8.5) were normalized as 100%, respectively. The activity determination was performed in triplicate, and error bounds represent ± sd.

**
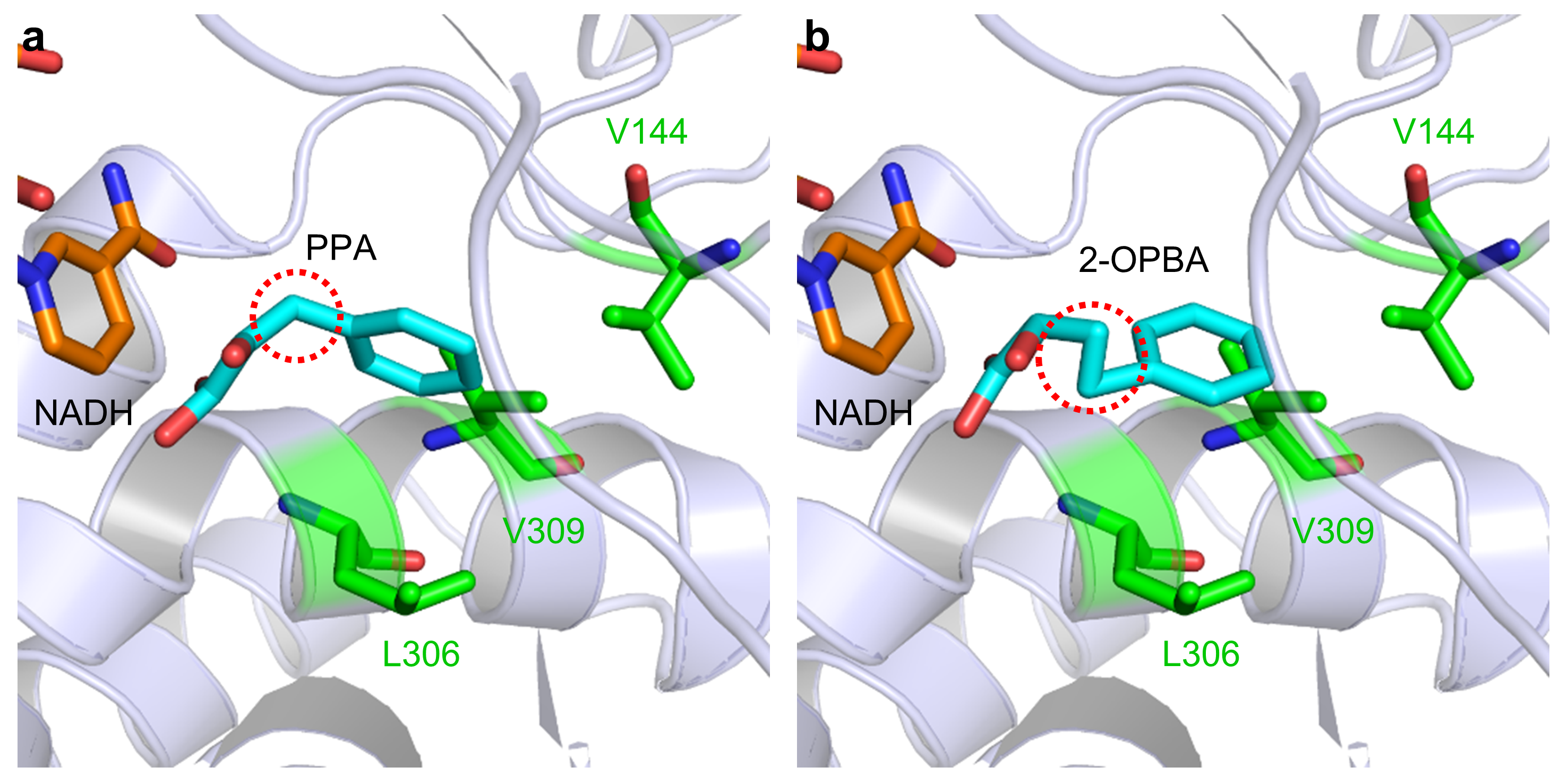
**

**Figure S8.** **Binding poses of native substrate PPA (a) and bulky substrate 2-OPBA (b) with** ***Bb*PheDH.** Structural homology model of *Bb*PheDH is shown in light blue cartoon representation. 2-OPBA is shown as cyan sticks. Focal residues V144, L306, and V309 are shown as yellow sticks. Coenzyme NADH is shown as orange sticks.


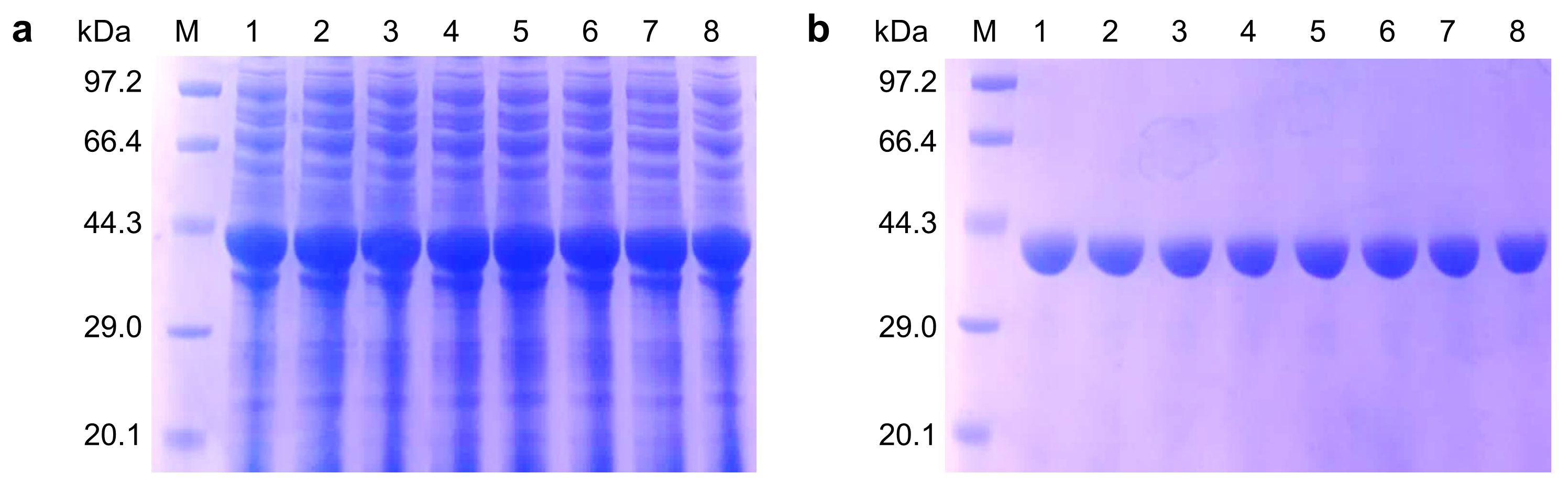


**Figure S9. SDS-PAGE analysis of *Bb*PheDH and its superior mutants.** Crude cell extract **(a)** and purified enzymes of *Bb*PheDH and its superior mutants **(b)**. **A**: Lane M, molecular weight marker, Lane 1 ~ 8, the crude cell extract of *Bb*PheDH, M1-1, M1-2, M1-3, M2-1, M2-2, M3-1, and M3-2, respectively. **B**: Lane M, molecular weight marker, Lane 1 ~ 8, purified enzymes of *Bb*PheDH, M1-1, M1-2, M1-3, M2-1, M2-2, M3-1, and M3-2, respectively.
